# Rating the quality of a body of evidence on the effectiveness of health and social interventions: A systematic review and mapping of evidence domains

**DOI:** 10.1002/jrsm.1290

**Published:** 2018-03-02

**Authors:** Ani Movsisyan, Jane Dennis, Eva Rehfuess, Sean Grant, Paul Montgomery

**Affiliations:** ^1^ Department of Social Policy and Intervention University of Oxford Oxford OX1 2ER UK; ^2^ London School of Hygiene and Tropical Medicine London WC1E 7HT UK; ^3^ Institute for Medical Informatics, Biometry and Epidemiology Ludwig‐Maximilians‐University Munich 81377 Germany; ^4^ RAND Corporation Santa Monica CA 90407‐2138 USA; ^5^ Department of Social Policy, Sociology and Criminology University of Birmingham Edgbaston Birmingham B15 2TT UK

**Keywords:** evidence rating, GRADE, guideline, intervention effectiveness, public health, systematic review

## Abstract

**Introduction:**

Rating the quality of a body of evidence is an increasingly common component of research syntheses on intervention effectiveness. This study sought to identify and examine existing systems for rating the quality of a body of evidence on the effectiveness of health and social interventions.

**Methods:**

We used a multicomponent search strategy to search for full‐length reports of systems for rating the quality of a body of evidence on the effectiveness of health and social interventions published in English from 1995 onward. Two independent reviewers extracted data from each eligible system on the evidence domains included, as well as the development and dissemination processes for each system.

**Results:**

Seventeen systems met our eligibility criteria. Across systems, we identified 13 discrete evidence domains: study design, study execution, consistency, measures of precision, directness, publication bias, magnitude of effect, dose‐response, plausible confounding, analogy, robustness, applicability, and coherence. We found little reporting of rigorous procedures in the development and dissemination of evidence rating systems.

**Conclusion:**

We identified 17 systems for rating the quality of a body of evidence on intervention effectiveness across health and social policy. Existing systems vary greatly in the domains they include and how they operationalize domains, and most have important limitations in their development and dissemination. The construct of the quality of the body of evidence was defined in a few systems largely extending the Grading of Recommendations Assessment, Development, and Evaluation approach. Grading of Recommendations Assessment, Development, and Evaluation was found to be unique in its comprehensive guidance, rigorous development, and dissemination strategy.

## 1 INTRODUCTION

Rating the quality of a body of evidence is an increasingly common component of systematic reviews and practice guidelines on intervention effectiveness. While assessing risks of bias in each *individual* study included in a research synthesis is an important and well‐established practice,^1^, ^2^ rating the quality of a body of evidence is a comparatively new practice that indicates the credibility and trustworthiness of the *totality* of evidence across studies in relation to a specific research question.^3^, ^4^ Systems for rating the quality of a body of evidence have been predominantly discussed and applied in health‐related systematic reviews and clinical guideline development.^5^, ^6^ The Cochrane Collaboration was the first organization to attempt to integrate the rating of a body of evidence as a mandatory procedure in research syntheses on intervention effectiveness. Specifically, Cochrane mandated use of the Grading of Recommendations Assessment, Development, and Evaluation (GRADE) approach in the conduct of Cochrane intervention reviews.^4^ Over the last decade, GRADE and other approaches for rating the quality of a body of evidence have proliferated. The GRADE approach, specifically, is currently used by over 100 organizations worldwide.^7^

Systems for rating the quality of a body of evidence typically involve an examination of various characteristics of evidence that ultimately results in a rating of that body of evidence. For example, in the GRADE approach, the process of rating starts with a consideration of the designs of included studies: If the body of evidence contributing to an outcome consists of randomized controlled trials (RCTs), the quality of a body of evidence is initially given a rating of “high,” while a body of evidence consisting of nonrandomized studies is initially given a rating of “low.”^8^ The body of evidence is then assessed by considering 8 further domains. Assessments within 5 domains—risk of bias, indirectness, inconsistency, imprecision, and publication bias—are used to downgrade the initial rating. For a body of evidence consisting of nonrandomized studies, assessments within the 3 remaining domains—magnitude of the effect, dose‐response relationship in the effect, and counteracting plausible residual bias or confounding—may be used to upgrade the initial “low” rating. Quality (“certainty” is also another frequently used term) of evidence is ultimately categorized into 1 of 4 ratings—high, moderate, low, and very low—that reflect the extent to which the review authors are confident or certain that an estimate of the effect for a specific outcome is correct.^8^

As use of evidence rating systems has increased, so have reports of challenges faced by those attempting to use these systems—particularly for research syntheses on social and public health interventions, which are often described as “complex.”^9^, ^10^, ^11^, ^12^ Interventions are viewed as complex for a variety of reasons. Some dimensions of complexity are ascribed to aspects of the interventions themselves,^13^, ^14^ such as interventions with multiple components that aim to address different and multiple causes of the problems (eg, both biological and social). Other dimensions of complexity are seen as emanating from system properties,^15^ that is to say, long, nonlinear, and dynamic relationships between interventions and outcomes, interactions and interdependencies between different components of interventions, and levels of target.^16^ Consideration of complexity may require additional guidance when rating the quality of a body of evidence.^11^, ^12^, ^17^ Study design is often a key issue, given that RCTs are not feasible or appropriate for many population‐level interventions. In addition, many researchers acknowledge that multifaceted heterogeneity between studies in systematic reviews of complex interventions is a more difficult type of problem and requires specific procedures of planning and analysis.^18^ There are also concerns that narrow perspectives on evidence synthesis, and the process of rating the quality of a body of evidence with simple hypotheses about the causal relationships may result in naïve and misleading synthesis results.^19^, ^20^ Furthermore, there are ambiguities around how best to conceptualize and interpret the construct of the quality of the body of evidence on the effectiveness of an intervention, when effects are contingent upon intervention programming, implementation, and contextual factors.^12^

### 1.1 Objectives

In view of the challenges reported in applying quality of a body of evidence rating outside of biomedical settings and interventions,^11^, ^12^ this paper sets out to systematically review systems for rating the quality of a body of evidence on intervention effectiveness, including systems from health and social policy. Previous systematic reviews investigating evidence rating systems have mainly focused on scientific evidence in biomedical contexts and have not included systems from social policy domains such as public health, education, and crime and justice.^21^, ^22^

The key objectives of this systematic review therefore are to (1) identify existing systems for rating the quality of a body of evidence on intervention effectiveness across health and social policy, (2) examine how these systems describe the construct of the quality of a body of evidence and map out discrete domains they use to rate that quality, and (3) describe the reported procedures used to develop and disseminate the systems.

The resultant “state of the field” map of the systems can be used by any reviewer to identify and adopt systems and domains for rating the quality of a body of evidence that are relevant for their specific needs.

## 2 METHODS

### 2.1 Eligibility criteria

Methods of this systematic review are described in detail in an a priori developed protocol (see Supporting Information). To be included in this review, a system had to (1) comprise a full‐length document reporting a procedure for rating the quality of a *body of evidence*, derived from evidence synthesis that integrates results across individual studies on the effectiveness of health or social interventions, and (2) be published in English from 1995 onward, when evidence rating was first proposed as a stage of research synthesis.^23^ Where a document discussed a system developed by others (eg, a literature review), we retrieved the original documents reporting those systems and examined them for eligibility. We excluded documents if they described a procedure for rating the quality of a body of evidence on intervention effectiveness for a specific clinical topic (eg, systems used in specific guidelines on osteoarthritis and brain injury), as these are largely covered by the 2 previous systematic reviews.^21^, ^22^ We also excluded systems that were no longer used by an organization (eg, the systems previously used by the Scottish Intercollegiate Guidelines Network and the Institute for Clinical Systems Improvement, before these organizations adopted the GRADE approach). Information on suspended use of these systems was either directly available on the organization's website or was obtained through email communication with representatives of the organization.

### 2.2 Systematic search strategy

We used a multicomponent search strategy with multiple sources in an attempt to maximize the sensitivity of the search. First, we updated search strategies used in previous systematic reviews^21^, ^22^ and expanded them to include social science databases. We ran these searches on June 2, 2016 in the following databases: Applied Social Sciences Index, Cochrane Methodology Register (Cochrane Library), EMBASE, MEDLINE, PsycINFO, SCIE Social Care Online, Scopus Social Sciences, and Social Sciences Citation Index (Web of Knowledge). Next, using the expertise of the authors and through bibliography searches of the related literature, we located and searched the websites of 83 key stakeholder organizations that specifically aim to aggregate, review, and assess evidence across social policy domains, such as child and family welfare, international development, crime and justice, public health, and education (see Supporting Information for the search strategy). Third, we searched the bibliographies of all the included documents and literature reviews containing secondary reporting of eligible systems. Finally, we consulted experts identified from the website searches to check whether we missed any systems.

Screening of all titles, abstracts, and full‐text documents was conducted by the first author (AM) by using the Rayyan web application for systematic reviews.^24^ A subset of randomly chosen titles (10%) was independently screened by a second author (JD). All discrepancies were discussed until agreement was reached.

### 2.3 Data extraction

We extracted data on 4 types of information. First, we extracted descriptive information about included systems, namely, the author, year, title, publication source, and eligibility criteria. We then extracted information from each system on how its authors defined the construct of the quality of a body of evidence. We further extracted details of specific domains within the system used to rate the quality of a body of evidence, how these domains were defined, and how ratings of the quality of a body of evidence were categorized (eg, “high,” “moderate,” or “low”). Extending the prespecified domains for development and dissemination of research reporting guidelines,^25^, ^26^ we looked at whether the systems reported any preparatory activities, such as a review of literature on existing domains for rating a body of evidence and consensus‐based activities, such as a Delphi exercise and expert meetings. Finally, we looked for information on how the documents describing the systems were written up and disseminated, such as whether the authors of the systems described how they planned to address criticism and feedback for the system or whether the system was available on an open‐access website.

The first author (AM) and a second independent reviewer (either JD or a research assistant) extracted information about the content, development, and dissemination of the included systems into a Microsoft Excel spreadsheet. Three independent reviewers (AM, JD, and ER) piloted the data extraction form on the same evidence rating system before continuing with the remaining systems.

### 2.4 Data synthesis

**Figure 1:**
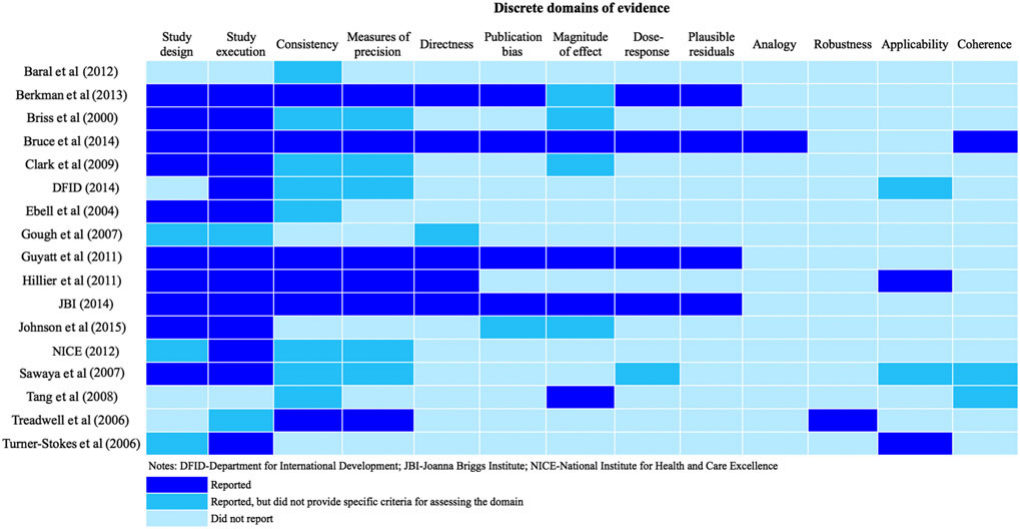
Reporting of the domains of evidence in the evidence rating systems for health and social interventions [Colour figure can be viewed at http://wileyonlinelibrary.com]

**Figure 2:**
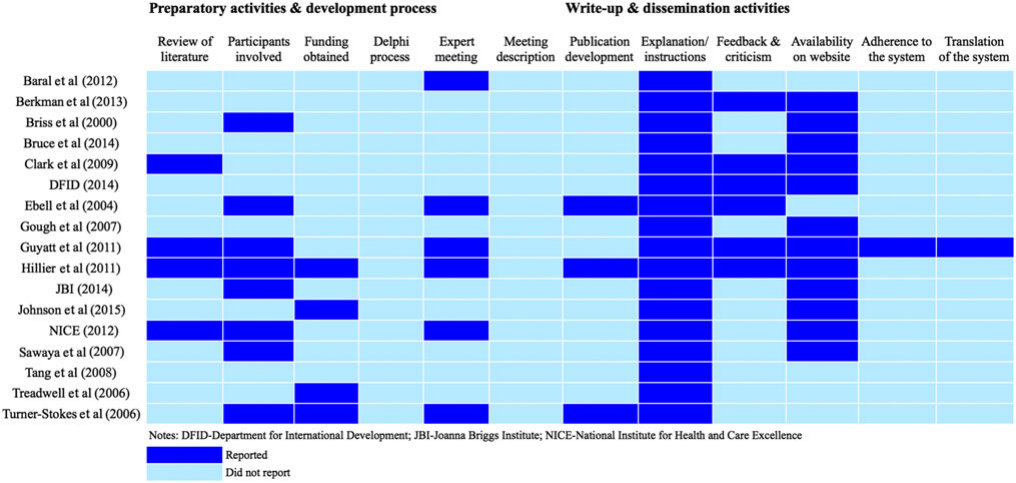
Reporting of the activities for developing and disseminating the evidence rating systems for health and social interventions [Colour figure can be viewed at http://wileyonlinelibrary.com]

We employed a 3‐step procedure to describe the domains of evidence for rating the quality of a body of evidence in the included systems. First, we created an inventory of all identified domains by using cross‐case tables.^27^ We examined these tables to compare how the domains for rating the quality of a body of evidence were labeled, defined, and operationalized across included systems. We then compiled a discrete (ie, nonredundant) list of domains of evidence considered in the included systems. The systems used different terminology to denote similar constructs and domains of evidence (for example, aspects of the domain that is termed as “imprecision” in the GRADE approach were covered by “precision” in the system used by the Agency for Healthcare Research and Quality [AHRQ] and fell under the domain termed “clinical impact” in the system adopted by the National Health and Medical Research Council of Australia). Where such overlap existed, we mainly followed the terminology of the GRADE approach to describe the discrete set of domains. We supplemented this with a list of additional domains that are not currently considered in the GRADE approach, but were found in other systems, and followed the terminology used in the systems to describe these domains. Finally, to help readers to visualize findings, we created a heat map summarizing how the systems reported the identified discrete domains of evidence (see Figure 1). By using different color shades, the heat map describes whether these domains of evidence are reported in each included system or not. Where a system reported the domain and yet did not provide specific criteria and guidance for rating it, the map denotes those as a different category of reporting (ie, with a brighter shade). Similar to this, we developed a second heat map describing how the authors reported activities underpinning the development and dissemination of the included systems (see Figure 2). Both of these heat maps were developed by first author (AM) and further verified by the second author (JD).

## 3 RESULTS

**Figure 3:**
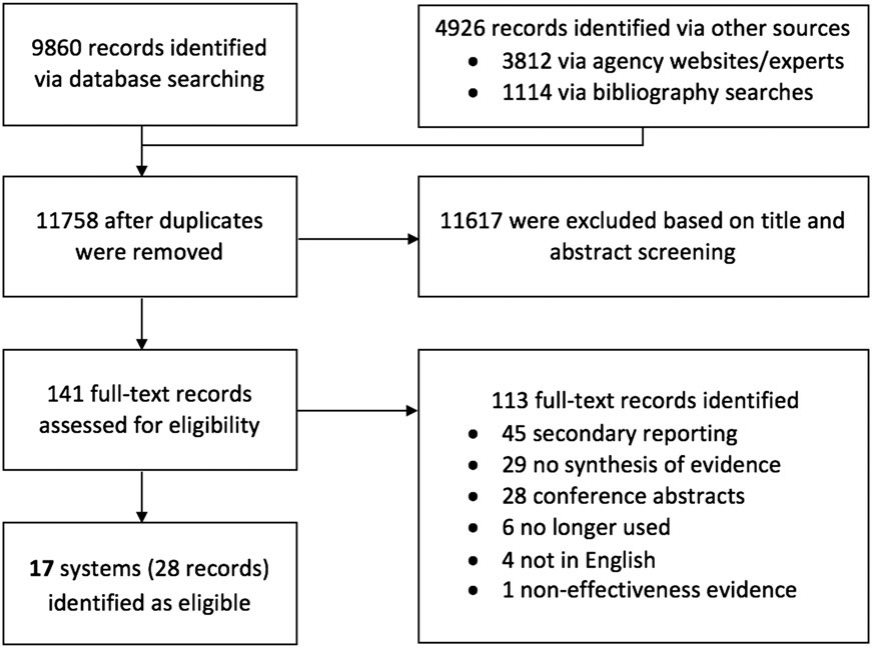
Systematic review PRISMA flow diagram

We identified 11,758 records after duplicates were removed. After title and abstract screening, we assessed the full texts of 141 records, from which 28 records were found to be eligible for inclusion in this review. Overall, these 28 records describe 17 evidence rating systems (see Figure 3 for the PRISMA flow diagram).

### 3.1 Excluded studies

Of the 113 records excluded at full text, 45 involved literature reviews of evidence rating systems, 28 were editorials or conference abstracts, and 4 records were not published in English (Chinese, French, Portuguese, and Spanish). Twenty‐nine records described procedures and domains for categorizing interventions on websites of different “what works” organizations, also known as evidence clearinghouses or evidence‐based program registers.^28^ Because these procedures and corresponding domains of evidence did not consider a “body of evidence,” we excluded them from this review (a full list of these systems and their specific domains are available from the first author upon request). Through website searches and contacts with experts, we established that 6 systems were no longer used.^23^, ^29^, ^30^, ^31^, ^32^, ^33^ A further system, the Confidence in the Evidence from Reviews of Qualitative Research, which is designed for sole application to a body of qualitative evidence, was not eligible for use in assessment of effectiveness evidence.^34^

### 3.2 Characteristics of the sample

Fourteen of the included systems were developed for healthcare, including general clinical and public health interventions (see Table 1). Only 3 systems were developed for other policy domains—specifically education, criminology, and international development.^35^, ^36^, ^37^ Three of the included systems were largely based on the GRADE approach^38^ but introduced modifications that warrant their classification as separate systems.^39^, ^40^, ^41^ Ten systems mentioned specific research synthesis methods for which the system was developed; most referred to a meta‐analysis or a “narrative synthesis” without a single pooled effect estimate^42^ to synthesize data on the effects of an intervention. Only 1 system was explicitly described for use with a mixed‐method approach to research synthesis.^36^ Eight of the systems described rating the quality of a body of evidence primarily within the context of research syntheses only, while 8 others described rating the quality of a body of evidence for a guideline development context. Only the GRADE approach addressed the conceptual and procedural differences when using the domains of evidence for assessing a body of evidence for research synthesis versus guideline development contexts.^38^

**Table 1:** Overview of the evidence rating systems for health and social interventions

Author (Year) Name of the System/Organisation	Domains of Evidence	Notes on the Domains of Evidence	Evidence Ratings	Evidence Synthesis Approach	Context of Application
Baral et al (2012)^58^ ***The highest attainable standard of evidence (HASTE)*** *“…focuses on triangulation of three distinct categories of evidence” (p. 572)*	1. Efficacy data 2. Implementation data^a^ 3. Plausibility data^a^	1. Consistent; inconsistent; limited 2. Consistent; inconsistent; limited^a^ 3. High; low; undefined^a^	• Grade 1 (strong) • Grade 2 (conditional) • Grade 2a (probable) • Grade 2b (possible) • Grade 2c (pending) • Grade 3 (insufficient) • Grade 4 (inappropriate)	Not specified	Guideline development in public health (specific focus on HIV/AIDs interventions)
Berkman et al (2013)^40^ ***Agency for Healthcare Research and Quality (AHRQ)*** *“…confidence in systematic review conclusions so that decision‐makers can use them effectively” (p. 1)*	1. Study design 2. Study limitations 3. Directness 4. Consistency 5. Precision 6. Reporting bias 7. Dose–response 8. Plausible confounding 9. Magnitude of effect 10. Applicability^a^	1. High (RCTs); low (non‐RCTs) 2. Risk of bias in RCTs/non‐RCTs 3. Divergence from the outcomes & comparisons of interest 4. Consistency in magnitude or direction 5. Sample size, width of 95% CI 6. Publication bias; selective outcome reporting bias; selective analysis reporting 7. Dose–response relationship 8. Counteracting confounding 9. Size of the estimate of the effect 10. Likelihood of expected results under the “real‐world” conditions^a^	• High • Moderate • Low • Insufficient	Quantitative: meta‐analysis or narrative synthesis	Evidence synthesis in clinical medicine
Briss et al (2000)^46^ ***The Guide to Community Preventive Services*** *“…confidence that changes in outcomes are attributable to the interventions”* *(p. 38)*	1. Design suitability 2. Quality of study execution 3. Number of studies 4. Consistent 5. Effect size 6. Applicability^a^ 7. Barriers to implementation^a^ 8. Economic evaluations^a^ 9. Other effects^a^	1. Greatest (concurrent comparison); moderate (comparison, but not concurrent); least (single group) 2. 6 categories of threats to validity: Good, fair or limited 3. – 4. Consistent in direction & size 5. Defined on a case‐by‐case basis 6. Applicability to local situations^a^ 7. – 8. – 9. Evidence on harms^a^	• Strong • Sufficient • Insufficient	Quantitative: meta‐analysis or narrative synthesis	Guideline development in public health
Bruce et al (2014)^41^ ***Grading of evidence for public health interventions (GEPHI)*** *“…it is useful to make a distinction between: (a) strength of evidence for causal inference, for which Bradford Hill viewpoints for distinguishing causation from association in environmental epidemiology are often referred to…, and (b) the quality of evidence for the intervention effect size (confidence in the estimate), for which GRADE may be used” (p. 11)*	1. Study design 2. Study limitations 3. Indirectness 4. Inconsistency 5. Imprecision 6. Reporting bias 7. Dose–response 8. Plausible confounding 9. Magnitude of effect 10. Analogy 11. Consistency 12. Coherence	1. High (RCTs); moderate (quasi‐experimental designs); low (other observational designs) 2. Risk of bias in RCTs/non‐RCTs 3. Divergence from the PICO elements 4. Heterogeneity in the effect estimates 5. Sample size, width of 95% CI 6. Failure to identify studies 7. Dose–response relationship 8. Counteracting plausibility 9. Size of the estimate of the effect 10. Supporting evidence with similar mechanisms 11. Consistent evidence across different settings 12. Coherence in the overall causal chain: High, moderate, weak (separate rating)	• High • Moderate • Low • Insufficient	Quantitative: meta‐analysis or narrative synthesis	Guideline development in public health
Clark et al (2009)^43^ ***Let evidence guide every new decision (LEGEND)*** *“The term ‘level’ was important to nurses to indicate the quality of an individual article; while ‘grade’ was more familiar to doctors and was adopted to indicate the quality of a body of evidence” (p. 1057)*	1. Study quality 2. Consistency 3. Number of studies	1. The aggregate quality ratings for individual studies (categorised based on an evidence hierarchy; e.g. 1a – good quality systematic review, 1b – lesser quality systematic review, 2a – good quality RCT/CCT; 2b – Lesser quality RCT/CCT; etc.) 2. The extent to which similar findings are reported: Yes; no; not available 3. –	• High • Moderate • Low • Grade‐not‐assignable	Not specified	Guideline development in clinical medicine
DFID (2014)^37^ ***Assessing the strength of evidence*** *“This note assumes that the overall ‘strength’ of a body of evidence is determined by the “avoidance of bias” of studies that constitute it, and by the size, context and consistency” (p. 3)*	1. Quality 2. Size of the body of evidence 3. Consistency 4. Context of the body of evidence	1. Assessed with regards to 7 domains: High; moderate; low 2. Large; medium small 3. Consistent; inconsistent; mixed 4. Global; context‐specific	• Very strong • Strong • Medium • Limited • No evidence	Not specified	Evidence synthesis in international development
Ebell et al (2004)^44^ ***Strength of recommendation taxonomy (SORT)*** *“We use the term level of evidence to refer to individual studies. The strength (or grade) of a recommendation for clinical practice is based on a body of evidence” (p. 60)*	1. Study quality 2. Consistency	1. Study quality (combined with study design considerations based on an evidence hierarchy) 2. Consistent; inconsistent	• Level 1 (good quality) • Level 2 (limited quality) • Level 3 (other evidence)	Quantitative: meta‐analysis	Guideline development in clinical medicine
Gough et al (2007)^35^ ***Weight of evidence: a framework for the appraisal of the quality and relevance of evidence*** *“Weight of evidence is a useful heuristic for considering how to make separate judgements on different generic and review specific criteria*” *(p. 11)*	1. Relevance of research design 2. Study execution 3. Relevance of the focus/context of evidence	1. A review specific judgement about the appropriateness of that form of evidence for answering the review question 2. Generally accepted criteria for evaluating the quality of evidence 3. A review specific judgement about the relevance of the focus of the evidence for the review question	• Weight of evidence A • Weight of evidence B • Weight of evidence C • Weight of evidence D	Not specified (different quantitative and qualitative approaches)	Evidence synthesis in education
Guyatt (2011)^38^ ***GRADE: Grading of recommendations assessment, development and evaluation*** *“The extent to which we can be confident that the estimates of effect are correct” (p. 394)*	1. Study design 2. Study limitations 3. Indirectness 4. Inconsistency 5. Imprecision 6. Publication bias 7. Dose–response 8. Plausible confounding 9. Magnitude of effect	1. High (RCTs); low (non‐RCTs) 2. Risk of bias in RCTs/non‐RCTs 3. Divergence from the PICO elements 4. Heterogeneity in effect estimates 5. Sample size, width of 95% CI 6. Failure to identify studies 7. Dose–response relationship 8. Counteracting confounding 9. Size of the estimate of the effect	• High • Moderate • Low • Very low	Quantitative: meta‐analysis or narrative synthesis	Evidence synthesis & guideline development in clinical medicine & public health
Hillier et al (2011)^48^ ***FORM: An Australian method for formulating and grading recommendations in evidence‐based guidelines*** *“…considering all of these elements across all of the research studies addressing the clinical question as a whole (the ‘body of evidence’)” (p. 2)*	1. Evidence base 2. Consistency 3. Clinical impact 4. Applicability 5. Generalisability	1. Quality; quantity and study design (evidence hierarchy: Level I – systematic reviews of RCTs; level II – RCTs, level III‐1 – Pseudorandomised trial; level III‐2 – Comparative study with concurrent control; level III‐3 – comparative study without concurrent controls) 2. Excellent; good; poor 3. Very large; substantial; moderate; slight 4. Excellent; good; satisfactory; poor 5. Excellent; good; satisfactory; poor	• A (evidence trusted) • B (evidence mostly trusted) • C (some support) • D (weak evidence)	Not specified	Guideline development in clinical medicine and public health
Joanna Briggs Institute (2014)^39^ ***Levels of evidence and grades of recommendations*** *“One of the main reason for continuing with Levels of Evidence system is to assist in assigning GRADE pre‐rankings…” (p. 4)*	1. Study design **2. The remaining domains of evidence follow those of the GRADE approach**	1. Level 1: Experimental; level 2: Quasi‐experimental; level 3: Observational‐analytic; level 4: Observational‐descriptive; level 5: Expert opinion	• High • Moderate • Low • Insufficient	Quantitative: meta‐analysis or narrative synthesis	Evidence synthesis in clinical medicine & public health
Johnson et al (2015)^36^ ***Introducing EMMIE: An evidence rating scale to encourage mixed‐method crime prevention synthesis*** *“…in addition to considering the extent to which evaluations manage to rule out biases that might distort estimates of effect size, we also need to gauge the extent to which they contribute to understanding of the contexts/moderators” (p. 462)*	1. Effects 2. Mechanisms/Mediators^a^ 3. Moderators/Contexts^a^ 4. Implementation^b^ 5. Economic analysis^a^	1. Consideration of evidence validity elements 2. Reference to and/or test of theory of change^a^ 3. Reference to and/or analysis of data relating to pre‐defined moderators^a^ 4. Account of implementation or implementation challenges^a^ 5. Estimation of marginal, total or opportunity costs^a^	Promotes descriptive profiles rather than a single overall score	Mixed‐method synthesis	Evidence synthesis in criminology
National Institute for Health and Care Excellence (NICE, 2012)^45^ ***Methods for the development of NICE public health guidance*** *“Strength of evidence – Reflecting the appropriateness of the study design to answer the question and the quality, quantity and consistency of evidence” (p. 89)*	1. Study design 2. Quality 3. Quantity 4. Consistency 5. Direction of the effect^a^ 6. Size of the effect^a^ 7. Applicability^a^	1. Appropriateness to answer the question 2. Assessment of both internal and external validity 3. – 4. – 5. Positive; negative; mixed; None^a^ 6. Small; medium; Large^a^ 7. Applicability of evidence in terms of PICO elements^a^	• No evidence • Weak evidence • Moderate evidence • Strong evidence • Inconsistent evidence	Quantitative: meta‐analysis or narrative synthesis Qualitative for questions other than intervention effectiveness	Guideline development in public health
Sawaya et al (2007)^47^ ***U.S. Preventive Services Task Force (USPSTF)*** *“The U.S. Preventive Services Task Force (USPSTF) defines certainty as “likelihood that the USPSTF assessment of the net benefit of a preventive service is correct” (p. 873)*	1. Study design 2. Study quality 3. Generalisability 4. Quantity 5. Consistency 6. Other	1. Evidence hierarchy (level I – RCT; level II‐1 – Controlled trial without randomisation; level II‐2 – Cohort and case–control; level II‐2 – Multiple time series; level III ‐ opinions) 2. Design specific: Good; fair; poor 3. – 4. – 5. – 6. Dose–response; fit within a biologic model	Chain of evidence: • High • Moderate • Low	Not specified	Guideline development in clinical medicine
Tang et al (2008)^57^ ***Grading of evidence of the effectiveness of health promotion interventions*** *“…the strength of evidence can be graded by using three criteria” (p. 832)*	1. Association 2. Repeatability 3. How it works	1. High (risk ratio of 2 or more) and statistically significant association: High, low, none 2. Reflects the consistency of the findings in different settings: Wide, limited, none 3. Reflects the known cause–effect mechanism for the intervention under study: Known; not known	• Grade 1 (strong) • Grade 2A (probable) • Grade 2B (possible) • Grade 2C (limited) • Grade 3 (insufficient)	Not specified	Evidence synthesis in public health
Treadwell et al (2006)^52^ ***A system for rating the stability and strength of medical evidence*** *“Our system draws a distinction between two types of conclusions: Quantitative and qualitative…a quantitative conclusion characterises the size of the effect, whereas a qualitative conclusion characterises the direction of the effect” (p. 6)*	1. Quality 2. Quantity 3. Informativeness 4. Homogeneity 5. Robustness	1. High; moderate; low; very low 2. Criterion met; criterion not met (at least 3 studies and 80% having calculable effect sizes) 3. Effect size 4. Homogeneous; heterogeneous 5. Tested through sensitivity analysis: Robust; not robust	• Strong • Moderate • weak • Inconclusive	Quantitative: meta‐analysis	Evidence synthesis in clinical medicine
Turner‐Stokes et al (2006)^50^ ***Generating the evidence base for the National Service Framework for long term conditions: a new research typology*** *“Each individual recommendation is then given an overall ‘grade of research evidence’ rating of A, B or C based on the quality of all the evidence supporting it and how much of it was directly relevant” (p. 97)*	1. Type of evidence 2. Study quality 3. Applicability	1. Primary research‐based; secondary research‐based; review‐based (no classification based on an evidence hierarchy) 2. Quality is assessed on the basis of 5 questions to reach a max. Score of 10 (includes a question on the appropriateness of the study design) 3. Population context of the study: Direct; indirect	• GRADE A • GRADE B • GRADE C	Quantitative: meta‐analysis or narrative synthesis Qualitative for questions other than intervention effectiveness	Evidence synthesis in clinical medicine & public health

a These domains of evidence go beyond rating the quality of a body of evidence on intervention effectiveness and are used in systems to further inform grading of the recommendations for practice. In the GRADE approach, these domains are separately specified as “Evidence to Decision” criteria (see Alonso‐Coello et al., 2016).

Notes: CCT – controlled clinical trial; CI – confidence interval; DFID – Department for International Development; PICO – population, intervention, comparison, outcomes; RCT – randomised controlled trial;

We identified inconsistencies in how included systems labeled and defined the rating of the quality of a body of evidence overall and the components of that rating. The most frequently used terms to describe the overall rating of the quality of a body of evidence were *strength of evidence*, *grades of evidence*, *quality*, *confidence*, or *certainty in evidence*.^37^, ^38^, ^40^, ^43^, ^44^, ^45^, ^46^, ^47^ In contrast, the most commonly used terms for assessing the conduct of individual included studies were *levels of evidence*, *critical appraisal*, *quality appraisal*, *study limitations*, *risk of bias*, and *study quality*.^37^, ^43^, ^44^, ^48^ From these, terms such as *levels of evidence*, *risk of bias*, and *study limitations* were mainly discussed regarding assessing studies for bias and internal validity, while *study quality*, *quality appraisal*, and *critical appraisal* were used to denote study execution more broadly regarding eliminating threats to both internal and external validities.

### 3.3 Defining quality of a body of evidence

Only 6 systems—3 of which are largely based on the GRADE approach—provided a definition for the construct of the quality of a body of evidence on intervention effectiveness.^38^, ^39^, ^40^, ^41^, ^46^, ^47^ In a systematic review context, the GRADE approach and 3 derivative systems defined quality of a body of evidence as “*the extent of confidence that an estimate of the effect is correct*.”^38^, ^39^, ^40^ The Guide to Community Preventive Services defined quality of a body of evidence as “*confidence that changes in outcomes are attributable to the interventions*”^46^ and the U.S. Preventive Services Task Force (USPSTF) as the “*likelihood that the assessment of the net benefit* (*i*.*e*., *benefits minus harms*) *of a preventive service is correct*.”^47^ The USPSTF definition is similar to how the GRADE approach defines the overall quality of a body of evidence in the context of guideline development, when considering all important outcomes associated with the intervention, including harms. In this context, GRADE defines the overall quality of a body of evidence as “*the extent of confidence that an estimate of the effect is adequate to support a particular decision or recommendation*.”^49^

To assess the net benefit of a preventive service, the USPSTF system uses analytic frameworks, also called “*chain of evidence*” diagrams to map out the specific linkages in the overall chain of evidence that must be present for a preventive service to be considered effective.^47^ The system assesses the quality of a body of evidence for each separate linkage in the chain of evidence to draw conclusions about the overall effectiveness of a preventive service. This approach is very similar to that adopted by the GRADE‐modified Grading of Evidence for Public Health Interventions (GEPHI) system.^41^ In addition to rating the quality of a body of evidence for the estimates of the effect of an intervention (which corresponds to the approach described in GRADE), the GEPHI system suggests to also rate the quality for the overall causal chain of an intervention. This rating of the confidence in the overall causal chain of an intervention is referred to as *coherence of evidence* assessment in the GEPHI system.^41^

### 3.4 Mapping of evidence domains

The evidence domains used to rate the quality of a body of evidence were often similar in concept across systems yet different in how they were described and operationalized. We encourage readers to use Table 1 and Figure 1 as 2 complementary sources of information on the identified evidence rating systems to examine the discrepancies in labeling and describing evidence domains. Table 1 provides an overview of the domains of evidence as they are reported in the original studies, while Figure 1 maps the 13 discrete domains we identified in included systems and presents how they are reported in each of the included systems. More information on how the specific evidence domains were defined and operationalized in each system is presented in Supporting Information (Online Supplement). In the sections below, we briefly summarize the identified discrete set of domains of evidence (see Figure 1), as well as the reported activities underpinning the development and dissemination of these systems (see Figure 2).

#### 3.4.1 Study design

Twelve systems included an evidence domain related to the design of the individual studies constituting the body of evidence. All but 4 of these systems^35^, ^36^, ^45^, ^50^ described an “evidence hierarchy” approach that influenced how overall quality of a body of evidence was assessed. Procedurally, this entailed initially privileging a body of evidence from certain study designs (namely RCTs) as providing a higher quality (compared with other study designs) before assessing other evidence domains. While all systems with an evidence hierarchy approach placed evidence from RCTs at the top of this hierarchy, many further privileged specific nonrandomized study designs over others. For example, the system used by the Joanna Briggs Institute^39^ suggested initial ratings of quality depending on whether a body of evidence consists of experimental (Level 1), quasi‐experimental (Level 2), or observational studies (Level 3). Similarly, the GRADE‐modified GEPHI system for public health interventions recommends that a body of evidence consisting of nonrandomized studies with controls or before and after [uncontrolled] studies have an initial rating of “moderate” quality if these studies used methods to minimize selection bias and confounding.^41^

#### 3.4.2 Study execution

Fifteen systems included an evidence domain related to assessing how well studies constituting the body of evidence were executed to minimize threats to internal and external validities (also labeled as *quality of study execution*, *risk of bias*, *study limitations*, and *study quality*). In most instances, however, systems mainly included criteria to assess risks of bias or threats to the internal validity for assessing study execution. A few systems, however, also included specific criteria for assessing the generalizability of the study results, that is, criteria related to the external validity of the individual studies in the body of evidence.

Systems varied in how they operationalized assessment of study execution. Some systems used design‐specific criteria, such as checklists or signaling questions for appraising RCTs^36^, ^38^, ^40^, ^43^ or longitudinal studies.^43^ Most systems, however, described more generic criteria to assess study execution across various study designs included in the body of evidence.^37^, ^45^, ^46^, ^48^, ^50^

#### 3.4.3 Consistency

Fourteen systems included an evidence domain related to the consistency of evidence. Generally, systems defined consistency as “*the extent to which findings are similar across included studies*” in a body of evidence,^48^ usually in reference to the degree of similarity in the magnitude and/or direction of effect estimates. Most systems, however, did not report any specific criteria on how to rate consistency in the body of evidence. Only a few systems discussed specific procedures, such as statistical testing for heterogeneity to rate consistency in the body of evidence. The GRADE‐modified GEPHI approach distinguished between 2 types of consistency ratings^41^: The first type was identical to the domain of the GRADE approach termed as *inconsistency* and defined as “*assessment of statistical heterogeneity in the estimates of the effect*.”^51^ The second type of consistency rating was specified in the system as “consistency” assessment and was defined as presence of “*consistent evidence across a large number of settings*, *geographical locations and diverse epidemiological study designs*.” The system argued that the fact that an intervention effect is reproducible under highly variable conditions suggests reduced likelihood that the observed effect is attributable to confounding or bias.^41^ This can increase a reviewer's confidence in the body of evidence regarding the overall effectiveness of an intervention.

#### 3.4.4 Measures of precision

Eleven systems included an evidence domain that we have classified as relating to *measures of precision* of the body of evidence: ie, considerations of the impact that random error may have on effect estimates. Systems differed widely in the level of specification and sophistication they required for assessing precision of the body of evidence. For instance, many systems recommend only considering the number of studies in the body of evidence as a measure of precision^37^, ^43^, ^45^, ^46^, ^52^; however, only 1 of these systems specifies a threshold for the minimum number of studies to be included in the body of evidence.^52^ Furthermore, only the GRADE approach and its variants described specific criteria for assessing precision regarding the sufficiency of the sample size of the body of evidence.^38^, ^39^, ^40^, ^41^ These systems assessed sufficiency of the sample size relative to an “optimal information size”: ie, “*number of patients* (*for continuous outcomes*) *and events* (*for dichotomous outcomes*) *that would be needed to regard a body of evidence as having adequate power*.”^53^ In addition, these systems also considered the boundaries of confidence intervals for an effect estimate in relation to a null effect and a clinically important effect threshold to make an overall judgment about the precision of a body of evidence. The estimate of the effect of an intervention is judged to be less precise if the confidence interval is wide to include a null effect or a threshold, which is considered as clinically unimportant.^53^

#### 3.4.5 Directness

In general, the systems used concepts of *directness*, *applicability*, and *generalizability* of evidence interchangeably and inconsistently—often without providing clear definitions or specific criteria to guide the assessment.^35^, ^37^, ^47^, ^48^, ^50^ In addition, these terms were not necessarily used as synonyms across the systems. For example, the system endorsed by the National Health and Medical Research Council of Australia used the term “applicability” to address whether the body of evidence was relevant to the local context (including the organizational and cultural contexts), while the term generalizability was used to refer to how precisely a body of evidence answered a review or a guideline question in populations and settings of interest.^48^ To disentangle the discrepancies in the terminology, we have used the terminology of the GRADE approach, namely, “directness” of evidence, to describe the domains of evidence from the included systems related to the notion of comparability of the evidence to the original research question. We have identified 6 systems that used this domain of evidence to assess how directly the available evidence answers a review or a guideline question regarding Population, Intervention, Comparison, and Outcomes elements of the question.^35^, ^39^, ^40^, ^41^, ^48^, ^54^

#### 3.4.6 Publication bias

Five systems included *publication bias* as a domain for rating the quality of a body of evidence.^36^, ^39^, ^40^, ^41^, ^55^ All but 1 of these systems followed a definition of publication bias as used within the GRADE approach, that is, “*a failure to identify studies as a result of studies remaining unpublished or obscurely published*.”^55^ The system used by AHRQ, on the other hand, considered publication bias as only 1 type of potential bias within a broader domain of reporting biases, which was itself defined as a decision by authors or journals to report research findings based on their direction and magnitude of effect.^40^ Selective outcome reporting and selective analysis reporting were the other types of reporting biases described in this system.

#### 3.4.7 Magnitude of effect

We identified 7 systems, which included *magnitude of effect* as a distinct domain to rate the quality of a body of evidence on the effectiveness of health or social interventions.^36^, ^39^, ^40^, ^41^, ^43^, ^46^, ^56^, ^57^ However, only 4 of these systems specified the thresholds for what they considered to be a “large” magnitude of effect.^39^, ^41^, ^56^, ^57^ This predominantly included a relative risk greaten than 2, or less than 0.2, as suggested in the GRADE approach.^56^

#### 3.4.8 Dose‐response

Overall, 5 systems considered *dose*‐*response* as a distinct domain of evidence when rating the quality of a body of evidence on the effectiveness of health or social interventions.^39^, ^40^, ^41^, ^47^, ^56^, ^57^ The systems commonly defined dose‐response as a “*pattern of a larger effect with greater exposure to an intervention*.”^40^

#### 3.4.9 Plausible residuals

All systems that followed the structure of the GRADE approach (overall 4 systems, including GRADE itself) considered counteracting confounding, as a domain to upgrade the quality of a body of evidence, when a body of evidence is mainly composed of observational studies.^39^, ^40^, ^41^, ^56^ Two possibilities were commonly applied: “*if all plausible residual biases would diminish the observed effect*, *or if all plausible residual biases would suggest a spurious effect when no effect is observed*.”^56^

#### 3.4.10 Analogy

Only 1 system—the GEPHI system—included an evidence domain related to analogous evidence. The GEPHI system operationalized analogous evidence as supporting evidence from similar or “analogous” interventions that are known to operate through the same or similar mechanisms, which, if present, could lead to a higher quality of a body of evidence rating.^41^ In the context of WHO guidelines on indoor air quality, the system discusses the example of how certainty in the effects of household air pollution from solid fuel can be enhanced by strong empirical evidence about the effects of second‐hand or active smoking. In this example, both household air pollution and second‐hand or active smoking expose individuals to similar combustion mixtures and therefore are viewed as analogous pieces of evidence.^41^

#### 3.4.11 Robustness

Robustness of evidence was described as a domain to rate the quality of a body of evidence by one system.^52^ The system suggests that reviewers measure robustness of evidence through sensitivity analysis with a priori defined thresholds. For example, a reviewer may decide a priori that a threshold for robustness assessment is one in which “*confidence intervals of the last three cumulative*, *random‐effects meta‐analyses remain fully on the same side of zero after removing of the study with the smallest weight*.”^52^

#### 3.4.12 Applicability

Four systems described applicability as a domain of evidence measuring the extent to which evidence may be applicable in a specific context.^37^, ^47^, ^48^, ^50^ It is worth highlighting that we identified 3 additional systems,^40^, ^45^, ^46^ which considered applicability of evidence as a separate judgment when making recommendations for practice. In these systems, discussion of applicability was held separately from other domains of evidence, and largely within a context of guideline development. For example, the GRADE‐based system endorsed by AHRQ clearly separates judgments of directness of evidence from that of applicability assessment. In this system, directness of evidence is defined to express “*how closely the available measures an outcome of interest*” and relies on 2 judgments^40^: the directness of the employed outcomes (ie, whether the available evidence is in fact only a proxy for an ultimate outcomes of interest) and directness of comparisons (ie, whether evidence derives from head‐to‐head comparisons). Meanwhile, the system defines applicability as the external validity of the evidence base regarding different populations and is considered explicitly but separately from the overall rating of the quality of a body of evidence.^40^

#### 3.4.13 Coherence

Only 3 systems included an evidence domain related to assessing the coherence of the causal pathway of an intervention^41^, ^47^, ^57^: that is, related to the assessment of a theory of change or a mechanism whereby an intervention is expected to operate. The GEPHI system recommends assessing confidence in the overall causal pathway between an intervention and distal outcomes (referred to as rating of *coherence* of evidence) regarding the evidence informing each individual link in the causal pathway.^41^ It describes this domain specifically in the context of interventions that involve complex causal pathways, where evidence directly linking the intervention with the distal outcomes is frequently unavailable. Similarly, by using analytic frameworks, the USPTSF system rates certainty of evidence in the overall chain of evidence for a specific preventive service.^47^ The system described by Tang and colleagues (2008) included assessment of the known mechanisms of action as a domain of evidence for rating of the quality of a body of evidence: “*if the theoretical basis is not known*, *the strength of evidence will be less convincing*.”^57^

### 3.5 Development and dissemination of the evidence rating systems

Figure 2 describes how the authors report procedures underpinning the development and dissemination of the systems. Regarding the preparatory activities for developing the system, only 4 systems empirically demonstrated the need for developing a new evidence rating system by referring to a separate publication by the same research team providing a critical appraisal of existing systems.^38^, ^43^, ^45^, ^48^ More frequently, the systems reported participants involved in the development of the system, and only 4 systems described obtaining funding for developing the system.^36^, ^48^, ^50^, ^52^ None reported conducting a Delphi process to develop the system, and only 5 reported hosting an expert meeting. However, with the exception of the GRADE approach, these systems did not provide further details on how these meetings were organized.^38^, ^44^, ^48^, ^50^, ^58^ The GRADE Working Group, on the other hand, organizes annual meetings lasting 2 to 3 days, where members of the group have an opportunity to meet face‐to‐face and further discuss and develop and refine aspects of the GRADE methodology.^7^

Regarding the write‐up and dissemination activities, only 3 systems described how the publication introducing the system was developed,^44^, ^48^, ^50^ while instructions for using the systems were predominantly described in the same document that introduced it. In 6 instances, willingness to incorporate the feedback of users and update the systems was mentioned.^37^, ^38^, ^40^, ^43^, ^44^, ^48^ Finally, although most systems are available online, information regarding adherence to or translation of the systems was not reported for any system except for GRADE (further details on this can be found on the website of the GRADE Working Group).^7^ The GRADE approach was also unique in involving ongoing working groups aiming to continually advance and expand the applicability of its methodology in step with developments in the area of evidence synthesis and assessment.

## 4 DISCUSSION

### 4.1 “State of the field” map of evidence rating systems for health and social interventions

This systematic review set out to describe the content, development, and dissemination of the systems for rating the quality of a body of evidence on intervention effectiveness across health and social policies. The review identified 17 systems that have made useful contributions to rating the quality of a body of evidence in health and social research synthesis. While this review identified domains of evidence that were commonly reported across the systems, there was significant variation in the specifications for these domains. The systems used different terminology to denote similar constructs of evidence when rating the quality of a body of evidence. The systems also varied in how they operationalized the domains of evidence, that is, in whether they described specific criteria and provided guidance for assessing each domain in an operationalizable manner. This review also identified domains of evidence that were found only in a few systems (see Figure 1). In general, the discrete set of domains identified in our review can be viewed to largely follow the “viewpoints for causation” proposed by Sir Austin Bradford Hill,^59^ although the relative coverage of these criteria across the included systems varies. For example, domains of evidence that will correspond to the Hill's criteria of experiment (study design and study execution), strength of association (magnitude of effect), consistency, and dose‐response gradient have been reported more extensively in evidence rating systems. Meanwhile, our review found only 3 systems, which considered domains corresponding to the Bradford Hill viewpoints of plausibility and coherence of evidence, and only 1 system included a domain on the analogous evidence. This can partly be explained by the challenges of developing an operational framework in research synthesis to assess the evidence against these criteria, including the need to search and integrate different sources of evidence.^60^

As this systematic review aimed to consider evidence rating systems across health and social policies, the identified variation in the terminology and description of evidence domains may partly reflect how research synthesis and its practice differs across policy areas and types of interventions. One of the most contested topics in the discussions of the quality of a body of evidence relates to the hierarchy of evidence initially described in the paradigm of evidence‐based medicine as an approach to differentiate between weak and strong study designs for assessing intervention effectiveness.^61^ While different versions of the evidence hierarchy have been described in clinical medicine, all of them place study designs such as case series (considered relatively weaker in protecting against threats to internal validity) in the bottom of the hierarchy, followed by case‐control and cohort studies in the middle and RCTs at the top.^62^ As our findings demonstrate, this evidence hierarchy approach is still used in many evidence rating systems, and particularly those developed and employed in clinical medicine. The widely adopted GRADE approach also follows this approach by way of describing 2 broad categories of study designs as a starting point for the body‐of‐evidence rating process (RCT evidence is initially rated as “high” quality and non‐RCT evidence as “low” quality). By contrast, our findings show that systems which are used in broader policy areas, such as public health, tend to allow more flexibility for differentiating between the many types of non‐RCT designs within their constructions of evidence hierarchies (see section 3.4.1 and Table 1). This practice is commensurate with a view that quasi‐experimental approaches should be given appropriate provisions in evidence rating systems as valuable methods for making causal inferences for public health interventions.^63^

Consistency of the body of evidence was another frequently reported domain of evidence in the included systems. Our findings demonstrate that evidence rating systems currently conceptualize consistency as similarity in the magnitude and direction of effect estimates across studies (of same or similar design) included in the body of evidence. There are, however, concerns that this approach only partly reflects the central tenet of scientific method, specifically that findings are replicable across “a variety of situations and techniques.”^59^ From this perspective, there are suggestions for a broader interpretation of the consistency of evidence to also consider “triangulation of evidence” across different methodological approaches when arriving at overall conclusions about intervention effectiveness.^64^ Triangulation has been defined as integration of evidence from several different methodological approaches (different study designs and analytical approaches), which address the same underlying causal question, but which vary in key sources of potential bias (for example, multivariable regression, instrumental variables, and RCTs).^65^ The importance of evidence triangulation has been cogently argued in the context of public health interventions involving longer causal pathways and multiple targets and behaviors, such as smoking or alcohol consumption, which are difficult (or impossible) to evaluate with RCTs alone. When the results from different methodological approaches are consistent in that they all point to the same conclusion, this is argued to strengthen the confidence in the overall findings (see Lawlor et al., 2016).^65^ Our review identified only 1 system which extended the domain of consistency to consider evidence from different study designs.^41^ Its broad interpretation, which looks at evidence from different methodological approaches to inform the rating of the quality of a body of evidence, was unique within our findings (see section 3.4.3).

Our review identified very few instances where the systems provided a definition for the construct of the quality of the body of evidence (see section 3.3). The few reported definitions mainly focus on the confidence in a direct estimate of the effect of an intervention—a definition initially suggested by GRADE. It is worth noting here that the most recent publication of the GRADE Working Group clarifies this definition of the quality of a body of evidence based on a priori defined threshold and the context of the review.^66^The quality of a body of evidence is currently conceptualized to reflect the extent to which reviewers can be confident that “*the true effect for a specific outcome lies on one side of a specified threshold or within a chosen range*.”^66^ The revised guidance suggests 3 types of ratings: noncontextualized, partly contextualized, and fully contextualized (see Table 2 for more details). In this new conceptualization, the quality of a body of evidence ratings is explicitly acknowledged to be contingent upon a priori defined thresholds of what may be considered as meaningful effects in different contexts. These thresholds and the resultant ratings may therefore vary depending on the context and purpose of the review.

**Table 2:** Approaches to defining certainty of evidence in GRADE (adapted from Hultcrantz et al)^66^

Setting	Degree of Contextualization	Threshold or Range	How to Set	What Certainty Rating Represents
Primarily for systematic reviews and health technology assessment	Noncontextualized	Range: 95% CI	Using existing limits of the 95% CI	Certainty that the effect lies within the confidence interval
		OR ≠ 1; RR ≠ 1; HR ≠ 1; RD ≠ 0	Using the threshold of null effect	Certainty that the effect of one treatment differs from another
Primarily for systematic reviews and health technology assessment	Partly contextualized	Specified magnitude of effect	eg, small effect is the effect small enough to not use the intervention if adverse effects/costs are appreciable	Certainty in a specified magnitude of effect for 1 outcome (eg, trivial, small, moderate, or large)
Primarily for practice guidelines	Fully contextualized	Threshold determined with consideration of all critical outcomes	Considering the range of effects on all critical outcomes and the values and preferences	Confidence that the direction of the net effect will not differ from 1 end of the certainty range to the other

Notes: CI indicates confidence interval; GRADE, Grading of Recommendations Assessment, Development, and Evaluation; HR, hazard ratio; OR, odds ratio; RD, risk difference; RR, risk ratio.

Regarding the activities underpinning the development and dissemination of the included systems, our review found that most systems did not report a comprehensive literature review or a consensus‐based procedure for developing the system (see Figure 2). In a similar vein, we found little reporting of how these systems were written up and further disseminated. It therefore remains difficult to assess how the described domains of evidence have been conceptualized and the degree to which they are, or are not, the product of scientific consensus. In the meantime, if not properly developed and disseminated, these systems may have limited value and use in research synthesis.^26^ In this regard, our review shows that the GRADE approach is 1 of the most comprehensive and transparent evidence rating systems in its guidance as well as its development and dissemination.^7^

### 4.2 Strengths and limitations

This review's unique contribution may lie in its thorough exploration of the content, development, and dissemination of the existing systems for rating the quality of a body of evidence across a range of policy areas, following systematic searches of bibliographic databases and sources of gray literature. Consequently, this review provides a comprehensive inventory of evidence domains considered when assessing quality of a body of evidence in research syntheses on intervention effectiveness across not just health, but social policy as well. Considering the acknowledged challenges associated with locating evidence rating systems through formal literature searches,^22^ we decided to balance the searches of scientific databases with an extensive search of gray literature, including 83 websites and databases of key stakeholder organizations. Furthermore, we complemented these searches with expert consultations to help locate these additional sources.

We note several limitations worth considering when interpreting our findings. First, we had to limit the scope of our review because of practical considerations. For instance, we included documents published in English only and therefore might have missed relevant work from the non‐English literature. Furthermore, given the identified variation in the terminology of the evidence domains, the mapping of these domains necessarily involved a degree of interpretation. It is therefore possible that another team of reviewers might have produced a different mapping of the domains with different conceptual categories. For example, another review team may have interpreted the broad evidence domain of the “efficacy data” of the Highest Attainable Standard of Evidence system^58^ as referring to the strength of association and therefore in the map classified under the category of the “measures of precision,” rather than consistency as we currently did. To address this concern, the initial mapping of evidence domains by the first author was independently verified by a second reviewer, and all issues were further discussed and clarified in the team.

### 4.3 Concluding remarks

The mapping of evidence domains presented in this review aims to clarify how domains of evidence for rating the quality of a body of evidence on intervention effectiveness have been specified, developed, and disseminated across health and social policies. We see 2 broad applications of our mapping of evidence domains. First, it can serve as an aid for researchers to help choose the evidence rating system and corresponding domains of evidence most suitable for their research focus and context of work. Second, by delineating important gaps in the content, development, and dissemination of current systems, it can indicate areas that may need further methodological development. It is worth noting that our mapping of domains should not be regarded as an expert advice on the best system for assessing the quality of a body of evidence on intervention effectiveness, but rather should be considered as a “state of the field” description and interpretation of the content and the processes of development and dissemination based on the information reported in the included systems.

## Supporting information

File S1. Review protocol
File S2. Specification of the evidence domains in the included evidence rating systems

## References

[1] Higgins JPT , Green S . Cochrane Handbook for Systematic Reviews of Interventions version 5.1.0. 2011 Retrieved from: http://www.handbook.cochrane.org/. Accessed May 23, 2017.

[2] Juni P , Altman DG , Egger M . Systematic reviews in health care: assessing the quality of controlled clinical trials. BMJ. 2001;323(7303):42‐46. 11440947 10.1136/bmj.323.7303.42 PMC1120670

[3] Guyatt GH , Oxman AD , Vist GE , et al. GRADE: an emerging consensus on rating quality of evidence and strength of recommendations. BMJ. 2008;336(7650):924‐926. 18436948 10.1136/bmj.39489.470347.AD PMC2335261

[4] Higgins J , Lasserson T , Chandler J , Tovey D , Churchill R . Methodological expectations of Cochrane interventions reviews. London: Cochrane; 2016.

[5] Gough D , Oliver S , Thomas J . An Introduction to Systematic Reviews. London, UK: SAGE Publications Ltd; 2012.

[6] Sackett DL . Evidence‐Based Medicine: How to Practive and Teach EBM. Edinburgh: Churchill Livingstone; 2000.

[7] The Grading of Recommendations Assessment, Development and Evaluation (GRADE) Working Group . Retrived from http://gradeworkinggroup.org/. Accessed November 17, 2017.

[8] Balshem H , Helfand M , Schunemann HJ , et al. GRADE guidelines: 3. Rating the quality of evidence. J Clin Epidemiol. 2011;64(4):401‐406. 21208779 10.1016/j.jclinepi.2010.07.015

[9] Barbui C , Dua T , van Ommeren M , et al. Challenges in developing evidence‐based recommendations using the GRADE approach: the case of mental, neurological, and substance use disorders. PLoS Med. 2010;7(8):10.1371/journal.pmed.1000322 PMC2930877 20824176

[10] Harder T , Abu Sin M , Bosch‐Capblanch X , et al. Towards a framework for evaluating and grading evidence in public health. Health Policy. 2015;119(6):732‐736. 25863647 10.1016/j.healthpol.2015.02.010

[11] Movsisyan A , Melendez‐Torres GJ , Montgomery P . Users identified challenges in applying GRADE to complex interventions and suggested an extension to GRADE. J Clin Epidemiol. 2016;70:191‐199. 26388040 10.1016/j.jclinepi.2015.09.010

[12] Rehfuess EA , Akl EA . Current experience with applying the GRADE approach to public health interventions: an empirical study. BMC Public Health. 2013;13(1):9. 23294803 10.1186/1471-2458-13-9 PMC3546302

[13] Craig P , Dieppe P , Macintyre S , Michie S , Nazareth I , Petticrew P . Developing and Evaluating Complex Interventions: New Guidance. Swindon: Medical Research Council; 2008.

[14] Lewin S , Hendry M , Chandler J , et al. Assessing the complexity of interventions within systematic reviews: development, content and use of a new tool (iCAT_SR). BMC Med Res Methodol. 2017;17(1):76 28446138 10.1186/s12874-017-0349-x PMC5406941

[15] Hawe P , Shiell A , Riley T . Theorising interventions as events in systems. Am J Community Psychol. 2009;43(3–4):267‐276. 19390961 10.1007/s10464-009-9229-9

[16] Diez Roux AV . Complex systems thinking and current impasses in health disparities research. Am J Public Health. 2011;101(9):1627‐1634. 21778505 10.2105/AJPH.2011.300149 PMC3154209

[17] Murad MH , Almasri J , Alsawas M , Farah W . Grading the quality of evidence in complex interventions: a guide for evidence‐based practitioners. Evid Based Med. 2016;22(1):20‐22. 27932400 10.1136/ebmed-2016-110577

[18] Pigott T , Shepperd S . Identifying, documenting, and examining heterogeneity in systematic reviews of complex interventions. J Clin Epidemiol. 2013;66(11):1244‐1250. 23953079 10.1016/j.jclinepi.2013.06.013

[19] Petticrew M . Time to rethink the systematic review catechism? Moving from ‘what works’ to ‘what happens’. Syst Rev. 2015;4(36):1‐6. 25875303 10.1186/s13643-015-0027-1 PMC4384311

[20] Petticrew M , Shemilt I , Lorenc T , et al. Alcohol advertising and public health: systems perspectives versus narrow perspectives. J Epidemiol Community Health. 2017;71(3):308‐312. 27789756 10.1136/jech-2016-207644

[21] Bai A , Shukla V , Bak G , Wells G . Quality Assessment Tools Project Report. Ottawa: Canadian Agency for Drugs and Technologies in Health; 2012.

[22] West S , King V , Carey T . Systems to Rate the Strength of Scientific Evidence. Evidence Report/Technology Assessment No. 47 (Prepared by the Research Triangle Institute‐University of North Carolina Evidence‐Based Practice Center Under Contract No. 290‐97‐0011). AHRQ Publication No. 02‐E016 Rockville, MD: Agency for Healthcare Research and Quality; 2002. PMC4781591 11979732

[23] Guyatt GH , Sackett DL , Sinclair JC , Hayward R , Cook DJ , Cook RJ . Users' guides to the medical literature. IX. A method for grading health care recommendations. Evidence‐based medicine working group. JAMA. 1995;274(22):1800‐1804. 7500513 10.1001/jama.274.22.1800

[24] Ouzzani M , Hammady H , Fedorowicz Z , Elmagarmid A . Rayyan—a Web and mobile app for systematic reviews. Syst Rev. 2016;5(1):210 27919275 10.1186/s13643-016-0384-4 PMC5139140

[25] Grant SP , Mayo‐Wilson E , Melendez‐Torres GJ , Montgomery P . Reporting quality of social and psychological intervention trials: a systematic review of reporting guidelines and trial publications. PLoS One. 2013;8(5):e65442 23734256 10.1371/journal.pone.0065442 PMC3666983

[26] Moher D , Schulz KF , Simera I , Altman DG . Guidance for developers of health research reporting guidelines. PLoS Med. 2010;7(2):e1000217 20169112 10.1371/journal.pmed.1000217 PMC2821895

[27] Miles BM , Huberman AM . Qualitative Data Analysis: An Expanded Sourcebook. Thousand Oaks, CA: Sage; 1994.

[28] Burkhardt JT , Schröter DC , Magura S , Means SN , Coryn CL . An overview of evidence‐based program registers (EBPRs) for behavioral health. Eval Program Plann. 2015;48:92‐99. 25450777 10.1016/j.evalprogplan.2014.09.006 PMC4413923

[29] Eccles M , Freemantle N , Mason J . North of England evidence based guidelines development project: methods of developing guidelines for efficient drug use in primary care. BMJ. 1998;316(7139):1232‐1235. 9553004 10.1136/bmj.316.7139.1232 PMC1112989

[30] Greer N , Mosser G , Logan G , Halaas GW . A practical approach to evidence grading. Jt Comm J Qual Improv. 2000;26(12):700‐712. 11143209 10.1016/s1070-3241(00)26059-8

[31] Harbour R , Miller J . A new system for grading recommendations in evidence based guidelines. BMJ. 2001;323(7308):334‐336. 11498496 10.1136/bmj.323.7308.334 PMC1120936

[32] Liddle J , Williamson M , Irwig L . Method for Evaluating Research and Guideline Evidence. Sydney, Australia: NSW Health Department; 1996.

[33] Weightman A , Ellis S , Cullum A , Sander L , Turley R . Grading Evidence and Recommendations for Public Health Interventions: Developing and Piloting a Framework. London: Health Development Agency; Support Unit for Research Evidence (SURE), Information Services, Cardiff University; 2005.

[34] Lewin S , Glenton C , Munthe‐Kaas H , et al. Using qualitative evidence in decision making for health and social interventions: an approach to assess confidence in findings from qualitative evidence syntheses (GRADE‐CERQual). PLoS Med. 2015;12(10):e1001895. 10.1371/journal.pmed.1001895 PMC4624425 26506244

[35] Gough D . Weight of evidence: a framework for the appraisal of the quality and relevance of evidence. Research Papers in Education. 2007;22(2):213‐228.

[36] Johnson SD , Tilley N , Bowers KJ . Introducing EMMIE: an evidence rating scale to encourage mixed‐method crime prevention synthesis reviews. J Exp Criminol. 2015;11(3):459‐473.

[37] DFID . How to Note: Assessing the Strength of Evidence. London: Department for International Development (DFID); 2014.

[38] Guyatt G , Oxman AD , Akl EA , et al. GRADE guidelines: 1. Introduction‐GRADE evidence profiles and summary of findings tables. J Clin Epidemiol. 2011;64(4):383‐394. 21195583 10.1016/j.jclinepi.2010.04.026

[39] Joanna Briggs Institute . Supporting document for the Joanna Briggs Institute levels of evidence and grades of recommendations. 2014 The Joanna Briggs Institute Levels of Evidence and Grades of Recommendations Working Party. Retrieved from: https://joannabriggs.org/assets/docs/approach/Levels-of-Evidence-SupportingDocuments-v2.pdf. Accessed October 26, 2016.

[40] Berkman ND , Lohr K , Ansari M , et al. Grading the Strength of a Body of Evidence When Assessing Health Care Interventions for the Effective Health Care Program of the Agency for Healthcare Research and Quality: An Update. AHRQ Publication No. 13(14)‐EHC130‐EF Rockville, MD: Agency for Healthcare Research and Quality; 2013. 24404627

[41] Bruce N , Pruss‐Ustun A , Pope D , Heather A , Rehfuess E . WHO indoor air quality guidelines: household fuel combustion: methods used for evidence assessment. World Health Organization (WHO) guidelines: household fuel combustion‐technical paper on evidence review methods; 2014.

[42] Popay J , Roberts H , Sowden A , et al. Guidance on the Conduct of Narrative Synthesis in Systematic Reviews: A Product From the ESRC Methods Programme. Lancaster, UK: Lancaster University; 2006.

[43] Clark E , Burkett K , Stanko‐Lopp D . Let Evidence Guide Every New Decision (LEGEND): an evidence evaluation system for point‐of‐care clinicians and guideline development teams. J Eval Clin Pract. 2009;15(6):1054‐1060. 20367705 10.1111/j.1365-2753.2009.01314.x

[44] Ebell MH , Siwek J , Weiss BD , et al. Strength of Recommendation Taxonomy (SORT): a patient‐centered approach to grading evidence in the medical literature. Am Fam Physician. 2004;69(3):548‐556. 14971837

[45] NICE . Methods for the Development of NICE Public Health Guidance: Process and Methods Guides. London: National Institute for Health and Care Excellence (NICE); 2012. 27905711

[46] Briss PA , Zaza S , Pappaioanou M , et al. Developing an evidence‐based guide to community preventive services‐methods. The task force on community preventive services. Am J Prev Med. 2000;18(1 Suppl):35‐43. 10806978 10.1016/s0749-3797(99)00119-1

[47] Sawaya GF , Guirguis‐Blake J , LeFevre M , Harris R , Petitti D , Force USPST . Update on the methods of the U.S. Preventive Services Task Force: estimating certainty and magnitude of net benefit. Ann Intern Med. 2007;147(12):871‐875. 18087058 10.7326/0003-4819-147-12-200712180-00007

[48] Hillier S , Grimmer‐Somers K , Merlin T , et al. FORM: an Australian method for formulating and grading recommendations in evidence‐based clinical guidelines. BMC Med Res Methodol. 2011;11:23 21356039 10.1186/1471-2288-11-23 PMC3053308

[49] Guyatt G , Oxman AD , Sultan S , et al. GRADE guidelines: 11. Making an overall rating of confidence in effect estimates for a single outcome and for all outcomes. J Clin Epidemiol. 2013;66(2):151‐157. 22542023 10.1016/j.jclinepi.2012.01.006

[50] Turner‐Stokes L , Harding R , Sergeant J , Lupton C , McPherson K . Generating the evidence base for the National Service Framework for long term conditions: a new research typology. Clin Med (Lond). 2006;6(1):91‐97. 16521364 10.7861/clinmedicine.6-1-91 PMC4954443

[51] Guyatt GH , Oxman AD , Kunz R , et al. GRADE guidelines: 7. Rating the quality of evidence‐inconsistency. J Clin Epidemiol. 2011;64(12):1294‐1302. 21803546 10.1016/j.jclinepi.2011.03.017

[52] Treadwell JR , Tregear SJ , Reston JT , Turkelson CM . A system for rating the stability and strength of medical evidence. BMC Med Res Methodol. 2006;6(1):52 17052350 10.1186/1471-2288-6-52 PMC1624842

[53] Guyatt GH , Oxman AD , Kunz R , et al. GRADE guidelines 6. Rating the quality of evidence‐imprecision. J Clin Epidemiol. 2011;64(12):1283‐1293. 21839614 10.1016/j.jclinepi.2011.01.012

[54] Guyatt GH , Oxman AD , Kunz R , et al. GRADE guidelines: 8. Rating the quality of evidence‐indirectness. J Clin Epidemiol. 2011;64(12):1303‐1310. 21802903 10.1016/j.jclinepi.2011.04.014

[55] Guyatt GH , Oxman AD , Montori V , et al. GRADE guidelines: 5. Rating the quality of evidence‐publication bias. J Clin Epidemiol. 2011;64(12):1277‐1282. 21802904 10.1016/j.jclinepi.2011.01.011

[56] Guyatt GH , Oxman AD , Sultan S , et al. GRADE guidelines: 9. Rating up the quality of evidence. J Clin Epidemiol. 2011;64(12):1311‐1316. 21802902 10.1016/j.jclinepi.2011.06.004

[57] Tang KC , Choi BC , Beaglehole R . Grading of evidence of the effectiveness of health promotion interventions. J Epidemiol Community Health. 2008;62(9):832‐834. 18701736 10.1136/jech.2007.061366

[58] Baral SD , Wirtz A , Sifakis F , Johns B , Walker D , Beyrer C . The Highest Attainable Standard of Evidence (HASTE) for HIV/AIDS interventions: toward a public health approach to defining evidence. Public Health Rep. 2012;127(6):572‐584. 23115382 10.1177/003335491212700607 PMC3461350

[59] Hill AB . The environment and disease: association or causation? Proc R Soc Med. 1965;58:295‐300. 14283879 10.1177/003591576505800503 PMC1898525

[60] Watson SI , Lilford RJ . Integrating multiple sources of evidence: a Bayesian perspective. In: Raine R, Fitzpatrick R, Barratt H, et al. Challenges, solutions and future directions in the evaluation of service innovations in health care and public health. Health Serv Deliv Res. 2016;4(16):1‐18.

[61] Guyatt G , Drummon R , Group E‐BMW . Users' Guide to the Medical Literature: A Manual for Evidence‐Based Clinical Practice. Chichago: American Medical Association; 2002.

[62] Murad MH , Asi N , Alsawas M , Alahdab F . New evidence pyramid. Evid Based Med. 2016;21(4):125‐127. 27339128 10.1136/ebmed-2016-110401 PMC4975798

[63] Geldsetzer P , Fawzi W . Quasi‐experimental study designs series‐paper 2: Complementary approaches to advancing global health knowledge. J Clin Epidemiol. 2017;89:12‐16. 28365307 10.1016/j.jclinepi.2017.03.015

[64] Vandenbroucke JP , Broadbent A , Pearce N . Causality and causal inference in epidemiology: the need for a pluralistic approach. Int J Epidemiol. 2016;45(6):1776‐1786. 26800751 10.1093/ije/dyv341 PMC5841832

[65] Lawlor DA , Tilling K , Davey Smith G . Triangulation in aetiological epidemiology. Int J Epidemiol. 2016;45(6):1866‐1886. 28108528 10.1093/ije/dyw314 PMC5841843

[66] Hultcrantz M , Rind D , Akl EA , et al. The GRADE Working Group clarifies the construct of certainty of evidence. J Clin Epidemiol. 2017;87:4‐13. 28529184 10.1016/j.jclinepi.2017.05.006 PMC6542664

