# Supplementary material for: Rating the quality of a body of evidence on the effectiveness of health and social interventions: A systematic review and mapping of evidence domains
Source: Res Synth Methods. 2018 Mar 2;9(2):224–42. doi: 10.1002/jrsm.1290 (PMC6001464; doi:10.1002/jrsm.1290)
Supplement: Supplementary file 1 — File S1. Review protocol File S2. Specification of the evidence domains in the included evidence rating systems [file JRSM-9-224-s001.zip › Supplementary File 1.docx]

**Supplementary File 1. Review Protocol**

**Domains for assessing the quality of a body of evidence on the effectiveness of health and social interventions: A systematic review protocol**

Ani Movsisyan^a^, Jane Dennis^b^, Eva Rehfuess^c^, Sean Grant^d^ & Paul Montgomery^e^

^a^Department of Social Policy and Intervention, University of Oxford, Oxford OX1 2ER, UK

^b^London School of Hygiene & Tropical Medicine, London WC1E 7HT, UK

^c^Institute for Medical Informatics, Biometry and Epidemiology, Ludwig-Maximilians-University, Munich 81377, Germany

^d^RAND Corporation, Santa Monica, CA 90407-2138, USA

^e^Department of Social Policy and Social Work, University of Birmingham, Birmingham B15 2TT, UK

Corresponding author:

Ani Movsisyan

Centre for Evidence Based Intervention

University of Oxford

Barnett House

32 Wellington Square

OX1 2ER

Phone: +44 (0) 1865 270325

Email: [ani.movsisyan@spi.ox.ac.uk](mailto:ani.movsisyan@spi.ox.ac.uk)

**Introduction**

Social and health problems and interventions to address those are the subject of scientific investigation in disciplines such as public health, international development, psychology, criminology, education and social work.^1^ Improvement of health and social outcomes conducive to human welfare is most frequently stated as the ultimate aim of these practice domains,^1^ which however often requires moving beyond strict disciplinary structures towards a transdisciplinary approach.^2^ The calls for transdisciplinary efforts to tackle social problems have been common in the recent decades, since social problems are increasingly viewed as embedded in complex dynamic systems of multiple agent interactions.^3,4^ From this perspective, health and social interventions are characterised as activities, strategies and techniques at either micro- (e.g. services) or macro-levels (e.g. policies) of implementation that operate by altering psycho-social processes, such as cognitions, behaviours, emotions, interpersonal relationships, structural or social aspects of environments.^5^ These mechanisms of change, i.e. the psycho-social processes whereby these interventions operate to produce social and related health outcomes have been described as the distinctive characteristics of social interventions.^5^

Health and social interventions and evaluation of their effectiveness are often characterised as both complicated and complex.^6,7^ Most frequently they involve multiple interacting components, difficult behaviours by those delivering or receiving the intervention, multiple targets and outcomes and certain degree of flexibility in their implementation, which complicates the evaluation of the effectiveness of these interventions when applying conventional methodologies.^8^ With the consideration of the complex dynamic system approach in evaluation research in the last decades, however, more recent scientific discussions draw on concepts from this approach, such as temporal dynamics, interactions, nonlinearity, and emergence to describe the complexity of health and social interventions and their evaluation.^7,9,10^ In this perspective, interventions are characterised as events in social systems of multiple agents, which through dynamic interactions transform capabilities and outcomes in the systems as holistic and emergent sum of these interactions.^11-13^ The consideration of complexity of social systems further challenges the use of traditional methods such as linear models in assessing intervention effectiveness as they may misrepresent the relationships between interventions and observed outcomes resulting from multiple interactions both within the programmatic aspects of the intervention (e.g. when they are comprised of multiple components), implementation process and wider contextual factors, as well as feedback loops and recursive nature of psycho-social processes^14-16^ (see Figure 1).

Commensurate with the principles of evidence-based practice (EBP), systematic reviews are considered to be the dominant method to judge the effectiveness of interventions through comprehensive search, synthesis and appraisal of evidence.^17^ Originally developed and applied in clinical medicine, the transfer of this method into social disciplines has not been straightforward.^18,19^ It has been shown that when used in wider public health and social interventions, the traditional systematic review method frequently results in conclusions of weak or mixed evidence, meanwhile struggling to capture the vast amount of evidence of different types to augment the information value of these reviews for practice decision-making.^20,21^Specific challenges have been reported in the narrow formulations and interpretations of review questions (i.e. the Population, Intervention, Comparator, Outcomes [PICO] framework), evidence synthesis and appraisal options focusing mainly on quantitative synthesis (i.e. meta-analysis) and internal validity of randomised controlled trials (RCTs).^16,22-24^ By way of illustration, recent scientific discussions suggest that instead of posing blanket questions in the form of whether interventions *work or do not work* (e.g. PICO framework), systematic reviews of public health and social interventions should aim to search and synthesise a range of evidence of *what happens when interventions are delivered in*
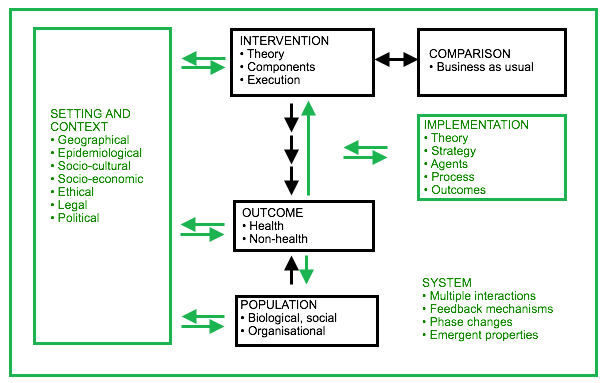
*various contexts and populations* to facilitate contextualised decision-making.^20,25^

**Figure 1.** Studying effectiveness of interventions in complex systems (taken from Dr. Eva Rehfuess)^26^

In this light, a number of projects have been launched in the recent years aiming to extend the boundaries of systematic reviews to address the complexity concerns and develop new techniques and frameworks, which will enable integration of different types of evidence to inform decisions on the effectiveness of these interventions. These include the Methodological Investigation of Cochrane Reviews of Complex Interventions (MICCI) aiming to understand different evidence synthesis options (e.g., integration of qualitative “trial sibling” and “unrelated” qualitative studies) and inform the development of a new chapter for the Cochrane Handbook on complex interventions,^27,28^ as well as the INTEGRATE Health Technology Assessment (INTEGRATE-HTA) project, which set out to develop concepts and methods for integrated assessment of complex health technologies, including assessment of effectiveness of complex interventions, context, setting and implementation.^29^

The Grading of Recommendations Assessment, Development and Evaluation (GRADE) approach is one of the most prominent systems for integrating and assessing the quality of the body of evidence to guide development of recommendations in clinical practices^30^. Currently, the GRADE approach is adopted by world-leading healthcare organisations such as the Cochrane and Campbell Collaborations, World Health Organization (WHO) and the US Agency for Healthcare Research and Quality (AHRQ), which investigate problems and interventions that extend beyond the boundaries of biomedical research and clinical settings and into social practice domains^31^. As in case of the systematic review method in general, the transition and application of the GRADE approach and its principles across social practice domains hasn’t been straightforward.

The common concerns of using the GRADE approach in wider health and social interventions are partly related to the abovementioned challenges of using the systematic review method as currently defined to collect and synthesise evidence on intervention effectiveness, but also include those specific to the assessment of the quality of the body of evidence in these interventions. These include inadequate definitions and criteria used to rate the quality of evidence, which are perceived to result in inappropriate downgrading of the effectiveness evidence in most cases.^32,33^ For example, the GRADE approach initially downgrades the quality of evidence for all types of observational studies no matter the arguments of many researchers from social disciplines that it might be the “best evidence possible” for these interventions, and that no further research will change the likelihood of finding different effects.^32^ Furthermore, researchers have asked for the addition of criteria in the GRADE approach to enable description of different types of evidence, which might be indirectly related to the intervention effectiveness, such as evidence on intervention implementation and context to augment the assessment of the quality of evidence when considering the complexity of social intervention.^32,34^ By corollary, researchers have called to redefine the quality of the body of evidence in the GRADE approach for social interventions.^34^ The GRADE approach defines the quality of the body of evidence as the extent of confidence that the estimate of effect is correct.^30^ However, as the effectiveness of health and social interventions is additionally influenced by a number of factors, including intervention programming, implementation and context (see Figure 1), an alternative interpretation has been suggested to define the quality of the body of evidence as *“the confidence that the effect is meaningful across a range of plausible implementation contexts”*.^34^

The foregoing challenges have hindered the uptake of the GRADE approach across wider social practice domains. For example, key organisations supporting principles of evidence-based practice in public health, such as the United State’s Community Guide of the Centre for Disease Control and Prevention, as well as the Public Health Guidance offered by the United Kingdom’s NICE Centre for Public Health Excellence have decided against the use of the GRADE approach and have opted for more *appropriate* systems and approaches that are deemed to better describe the evidence base of public health interventions.^34,35^

**Objectives**

Considering the reported challenges of using the GRADE approach outside of biomedical settings and interventions, the objective of this systematic review is to investigate the content, development and dissemination of the existing systems for grading the quality of a body of evidence on the effectiveness of health and social interventions. In literature these systems are often referred to as evidence grading systems. This “state of the field” map of the evidence grading systems can serve as an inventory for systematic reviewers to identify and choose the systems, which best suit their specific needs.

Previous systematic reviews that investigate evidence grading systems mainly focus on scientific evidence in biomedical practice contexts and not on those used in social practices. For example, a systematic review conducted by AHRQ in 2002 identified 40 systems for grading the quality of a body of evidence the majority of which were derived from clinical practice guideline literature.^36^ This review was updated and expanded in 2005 and 2007 as part of the Quality Assessment Tools (QAT) Project launched by the Canadian Agency for Drugs and Technologies in Health (CADTH).^37^ However, the latter effort also focused on evidence grading systems for use in clinical contexts as it was specifically conducted to support the goals of optimising the drug-related health outcomes and cost-effective use of drugs of the Canadian Optimal Medication Prescribing and Utilisation Service (COMPUS). Furthermore, neither of these reviews comprehensively explored the development and dissemination of the identified evidence grading systems.

**Methods**

***Inclusion Criteria***

In order to be eligible for inclusion in this review, an evidence grading system should be a published document reporting a procedure for rating the quality of a body of evidence on the effectiveness of a health and/or a social intervention (see the Introduction above on definitions of health and social interventions). For the purposes of this review, *a body of evidence will be defined as a totality of evidence synthesised across individual studies in relation to a specific question of intervention effectiveness*. For feasibility reasons, the search will be limited to documents available in English and published from 1995 onward, when the topic of rating the quality of a body of evidence came fore. If the same document introduces more than one evidence grading system, they will be included in the review as separate grading systems.

***Exclusion Criteria***

The following publications will be excluded from the review:

- Documents that provide general knowledge on or discussion of aspects of evidence-based practice, evidence synthesis, critical appraisal, systematic reviews and meta-analyses (e.g., best practices of systematic reviewing)
- Documents that discuss evidence grading systems developed by others. Where a publication discusses an evidence grading system developed by another person or group, the original document will be retrieved and examined to determine whether that system is eligible. The second order explanation will not be included in the review to avoid double counting (e.g., reviews of evidence grading systems).
- Documents that introduce methodologies for reporting or critical appraisal of specific primary research designs (e.g., reporting guidelines and quality assessment tools [QATs] for randomised controlled trials, observational studies, diagnostic test studies, systematic reviews, meta-analyses).
- Documents that introduce procedures for grading the quality of a body of evidence with a specific focus on biomedical interventions and problems (e.g. neck pain, brain injury), or unrelated to the evaluation of intervention effectiveness (e.g., methodologies that are specifically designed for grading evidence for risk factors, diagnostics, and a body of qualitative evidence).

***Search Strategy and Study Selection***

One of the acknowledged methodological challenges of previous reviews has been associated with locating evidence grading systems through formal literature searches. For example, the vast majority of the systems analysed in AHRQ review in 2002 were identified through bibliography searches and contacts with relevant experts.^36^ This challenge was mainly attributed to the lag in development of Medical Subject Headings (MeSH) specific for appropriate indexing of terms related to evidence-based practice and evidence assessment methods at the time. In order to overcome this challenge a QAT project review conducted in 2005 and updated in 2007 first ran a very sensitive search for systematic reviews of grading systems followed by a search for individual systems complemented by expert consultation.^37^ Considering the recommendations highlighted in the AHRQ report regarding the search strategy for evidence grading systems, this systematic review will consider expert consultation and searches in supplemental (grey) literature as a key strategy for locating relevant evidence grading systems. However, in light of the rapid development of the principles and concepts of EBP in the last decades, this review will also consider a formal literature search updating the search strategies from previous AHRQ and QAT project reviews and tailoring those to social science databases.

Another challenge for this review is the need to look at publications across social practice domains, as social interventions are subject to transdisciplinary investigation. The review will therefore adopt a multi-component search strategy as described below to enable efficient retrieval of relevant publications from multiple sources:

1. Systematic searches in the following scientific databases (see Appendix 1 below for specific search strings):

- Applied Social Sciences Index (ASSIA)
- EMBASE (Ovid)
- Cochrane Methodology Register (Cochrane Library)
- MEDLINE (Ovid)
- PsycINFO (Ovid)
- Scopus Social Sciences
- Social Sciences Citation Index (Web of Knowledge)
- SCIE Social Care Online

1. Searches on the websites of key stakeholder organisations (see Appendix 2 for a full list)
2. Consulting experts to ensure comprehensiveness of the search

- Experts will be located from the specialist websites and agencies listed in Appendix 2
- Experts will also be located through the network of the author team

1. Searches of bibliographies of all the eligible documents

***Data Screening, Management and Extraction***

After removal of duplicate studies, one reviewer (AM) will assess all titles and abstracts, removing those which are not relevant. This will be followed by assessment of full texts of those studies, which were deemed eligible after title/abstract screening. A subset of randomly chosen titles (10%) will be independently screened by a second reviewer (JD). Disagreement will be resolved by discussion, and where necessary by consulting a third review author.

Two review authors (AM and JD) will independently extract data from the included studies into the data extraction form. Inconsistencies between the two reviewers will be resolved by discussion, and where necessary by consulting a third review author.

Following the objectives of this review, data will be extracted on four types of information:

1. Descriptive information will be extracted about included systems, such as the author, year, title, publication source and eligibility criteria.
2. Information on the content of the included systems will be extracted, namely how the construct of the quality of a body of evidence is defined, what specific domains and criteria the systems use to rate the quality of a body of evidence, how these domains and criteria are defined, and how ratings are categorised (e.g., “high”, “moderate”, “low”).
3. To examine the development process of the included systems, information on the key development activities will be extracted based on the recommended techniques described by Moher and colleagues for developing research reporting guidelines.^38,39^
4. Finally, information on how the documents describing the systems were written and disseminated will be extracted following the key activities described by Moher et al. for writing and disseminating research reporting guidelines.^38,39^

The items of the data extraction form are provided in Appendix 3. These have been piloted on the same included system by three reviewers (AM, JD, and ER) to ensure that they allow accurate and reliable extraction of relevant data.

***Data Synthesis***

To enable a structured synthesis of collected data, a three-step procedure will be employed:

1. First, all identified domains will be listed in an inventory using cross-case tables.^40^ This will enable comparison of the systems on how they define, label and operationalise the domains of evidence for rating the quality of a body of evidence. A summary of each include system will be presented in a separate Table in the review.
2. Second, a discrete (i.e., non-redundant) list of domains will be compiled eliminating duplications and redundancies, and the relative coverage of these domains by the included systems will be illustrated diagrammatically.
3. Finally, another diagram will be constructed describing whether the included systems report the key development and dissemination activities as described by Moher and colleagues.^38^ These diagrams will be developed by two reviewers (AM and JD) working in collaboration.

**Acknowledgements**

We would like to acknowledge that this systematic review is conducted as part of the larger project aiming to develop a new [GRADE Guidance for Complex Interventions](https://www.birmingham.ac.uk/schools/social-policy/departments/social-policy-sociology-criminology/research/projects/2017/GRADE-Guidance-for-Complex-Social-Interventions.aspx). All the authors are current members of the GRADE Working Group. The funding for this project has been obtained from the Economic and Social Research Council (ES/N012267/1).

**References**

1. Fraser MW. *Intervention research: developing social programs.* New York; Oxford: Oxford University Press; 2009.

2. Gibbons M, Limoges C, Nowotny H, Schwatzman S, Scott P, Trow M. *The new production of knowledge: The dynamics of science and research in contemporary societies.* London: Sage Publications Ltd; 1994.

3. Satterfield JM, Spring B, Brownson RC, et al. Toward a transdisciplinary model of evidence-based practice. *Milbank Q.* 2009;87(2):368-390.

4. Miller JH, Page SE. *Complex adaptive systems: an introduction to computational models of social life.* Princeton, New Jersey: Princeton University Press; 2007.

5. Montgomery P, Grant S, Hopewell S, et al. Protocol for CONSORT-SPI: an extension for social and psychological interventions. *Implement Sci.* 2013;8:99.

6. Rogers P. Using programme theory to evaluate complicated and complex aspects of interventions. *Evaluation.* 2008;14(1):29-48.

7. Patton MQ. *Developmental evaluation: applying complexity concepts to enhance innovation and use.* New York: A Division of Guilford Publications, Inc.; 2011.

8. Craig P, Dieppe P, Macintyre S, Michie S, Nazareth I, Petticrew M. *Developing and evaluating complex interventions: new guidance* London: Medical Research Council; 2008.

9. Galea S, Riddle M, Kaplan GA. Causal thinking and complex system approaches in epidemiology. *Int J Epidemiol.* 2010;39(1):97-106.

10. Plsek PE, Greenhalgh T. Complexity science: The challenge of complexity in health care. *BMJ.* 2001;323(7313):625-628.

11. Datta J, Petticrew M. Challenges to evaluating complex interventions: a content analysis of published papers. *BMC Public Health.* 2013;13:568.

12. Hawe P, Shiell A, Riley T. Theorising interventions as events in systems. *Am J Community Psychol.* 2009;43(3-4):267-276.

13. Reed M, Harvey D, L. The new science and the old: Complexity and realism in the social sciences. *J Theor Soc Behav.* 1992;22(353-380).

14. Egan M, Bambra C, Petticrew M, Whitehead M. Reviewing evidence on complex social interventions: appraising implementation in systematic reviews of the health effects of organisational-level workplace interventions. *J Epidemiol Community Health.* 2009;63(1):4-11.

15. Petticrew M. When are complex interventions 'complex'? When are simple interventions 'simple'? *Eur J Public Health.* 2011;21(4):397-398.

16. Sanderson I. Making sense of "what works": Evidence based policy making as instrumental rationality? *Public Policy Admin.* 2002;17(3).

17. Lavis J, Posada F, Haines A, Osei E. Use of research to inform public policy-making. *Lancet.* 2004;364:1615-1621.

18. Petticrew M, Roberts H. Systematic reviews-do they 'work' in informing decision-making around health inequalities? *Health Econ Policy Law.* 2008;3:197-211.

19. Pawon R, Tilley N. *Realist evaluation.* London: Sage Publications Ltd; 1997.

20. Petticrew M. Time to rethink the systematic review catechism? Moving from 'what works' to 'what happens'. *Syst Rev.* 2015;4(1):36.

21. Threlfall AG, Meah S, Fischer AJ, Cookson R, Rutter H, Kelly MP. The appraisal of public health interventions: the use of theory. *J Public Health (Oxf).* 2015;37(1):166-171.

22. Anderson LM, Petticrew M, Chandler J, et al. Introducing a series of methodological articles on considering complexity in systematic reviews of interventions. *J Clin Epidemiol.* 2013;66(11):1205-1208.

23. Noyes J, Gough D, Lewin S, et al. A research and development agenda for systematic reviews that ask complex questions about complex interventions. *J Clin Epidemiol.* 2013;66(11):1262-1270.

24. Victora CG, Habicht JP, Bryce J. Evidence-based public health: moving beyond randomized trials. *Am J Public Health.* 2004;94(3):400-405.

25. Sanderson I. Complexity, "practical rationality" and evidence-based policy making. *Policy & Politics.* 2006;34(1):115-132.

26. Rehfuess E. *Adopting a systems perspective in public health: methodological implications and stakeholder engagement.* A plenary session at the 23^rd^ Cochrane Colloquium. Vienna, Austria; 2015.

27. Noyes J, Hendry M, Booth A, et al. Current use was established and Cochrane guidance on selection of social theories for systematic reviews of complex interventions was developed. *J Clin Epidemiol.* 2016;75:78-92.

28. Noyes J, Hendry M, Lewin S, Glenton C, Chandler J, Rashidian A. Qualitative "trial-sibling" studies and "unrelated" qualitative studies contributed to complex intervention reviews. *J Clin Epidemiol.* 2016;74:133-43.

29. INTEGRATE Health Technology Assessment (HTA) Project. Retrieved from http://www.integrate-hta.eu/. Accessed 5 April, 2016.

30. Guyatt G, Oxman A, D., Aklm E, A., et al. GRADE guidelines: 1. Introduction—GRADE evidence profiles and summary of findings tables. *J Clin Epidemiol.* 2011;64(4):383-394.

31. The Grading of Recommendations Assessment, Development and Evaluation (GRADE) Working Group. 2014. Retrieved from http://www.gradeworkinggroup.org/. Accessed October 22, 2014.

32. Movsisyan A, Melendez-Torres GJ, Montgomery P. Users identified challenges in applying GRADE to complex interventions and suggested an extension to GRADE. *J Clin Epidemiol.* 2016;70:191-199.

33. Movsisyan A, Melendez-Torres GJ, Montgomery P. Outcomes in systematic reviews of complex interventions never reached "high" GRADE ratings when compared to those of simple interventions. *J Clin Epidemiol.* 2016;78:22-33.

34. Rehfuess EA, Akl EA. Current experience with applying the GRADE approach to public health interventions: an empirical study. *BMC Public Health.* 2013;13:9.

35. The NICE public health guidance development process. 2012. Retrieved from http://www.nice.org.uk/aboutnice/howwework/developingnicepublichealthguidance/publichealthguidanceprocessandmethodguides/. Accessed December 21, 2014.

36. West S, King V, Carey TS., et al. *Systems to rate the strength of scientific evidence. Evidence Report/Technology Assessment No. 47 (Prepared by the Research Triangle Institute–University of North Carolina Evidence-based Practice Center under Contract No. 290-97-0011). AHRQ Publication No. 02-E016.* Rockville, MD: Agency for Healthcare Research and Quality;2002.

37. Bai A, Shukla VK., Bak G, Wells G. *Quality Assessment Tools Project Report.* Ottawa: Canadian Agency for Drugs and Technologies in Health; 2012.

38. Moher D, Schulz KF, Simera I, Altman DG. Guidance for developers of health research reporting guidelines. *PLoS Med.* 2010;7(2).

39. Grant SP, Mayo-Wilson E, Melendez-Torres GJ, Montgomery P. Reporting quality of social and psychological intervention trials: a systematic review of reporting guidelines and trial publications. *PLoS One.* 2013;8(5).

40. Miles BM, Huberman AM. *Qualitative data analysis: an expanded sourcebook.* 2nd ed. Thousand Oaks, CA: Sage; 1994.

**Appendix 1. Searches in the Scientific Databases**

Searches in the following databases were conducted on June 2, 2016.

1. **MEDLINE (Ovid Interface, 1946 to May Week 4, 2016)**

| **Search Number** | **Search String** | **Results** |
| --- | --- | --- |
| #1 | exp Research/ | 515677 |
| #2 | exp Research Design/ | 371918 |
| #3 | exp Guideline/ | 27792 |
| #4 | exp Epidemiologic Studies/ | 1909422 |
| #5 | exp Study Characteristics/ | 4473942 |
| #6 | Feasibility Studies/ | 50925 |
| #7 | Program Evaluation/ | 51432 |
| #8 | Evidence Based Medicine/ | 61064 |
| #9 | Human Experimentation/ | 10229 |
| #10 | Meta Analysis/ | 66825 |
| #11 | (systematic adj3 review$).mp. | 70050 |
| #12 | (meta-analys#s).mp. | 101003 |
| #13 | (evidence adj3 synthes#s).mp. | 3379 |
| #14 | or/#1 – #13 | 6395228 |
| #15 | ((approach or approaches or standard*1 or system*1 or instrument*1 or framework*1 or level*1 or hierarchy or hierarchies or method* or profile*1 or guideline*1 or guide*1) adj3 evidence).ti.^[[1]](#footnote-1)^ | 4498 |
| #16 | (quality or confidence or certainty or strength).tw. | 1028928 |
| #17 | ((rate or rates or rating or grade or grades or grading or measure or measuring or score or scoring or assess or assessing or evaluate or evaluating or tool*1 or checklist*1 or system*1 or instrument*1) adj3 ((level*1 or hierarchy or hierarchies) adj3 evidence)).tw. | 506 |
| #18 | #15 and #16 | 827 |
| #19 | #16 and #17 | 264 |
| #20 | ((grade or grades or grading or rate or rates or rating or evaluate or evaluating or evaluation or assess*) adj3 evidence).ti. | 1528 |
| #21 | ((grade or grades or grading or rate or rates or rating or evaluate or evaluating or assess or assessing or quality or strength) adj3 recommendation*1).ti. | 307 |
| #22 | ((framework*1 or system*1 or instrument*1) adj2 evidence).ti. | 419 |
| #23 | #18 or #19 or #20 or #21 or #22 | 3199 |
| #24 | ((grade or grades or grading or rate or rates or rating or assess* or evaluate or evaluating or appraisal or appraising) adj2 (quality or strength) adj5 (evidence or recommendation*1)).ti. | 108 |
| #25 | ((method or methods or methodology or approach or approaches) adj3 (assess or assessing or assessment or evaluate or evaluating) adj3 (recommendation*1 or evidence)).ti. | 43 |
| #26 | (good adj3 practi#e*1 adj3 recommendation*1).tw. | 229 |
| #27 | (#14 and #23) or #24 or #25 or 26 | 2385 |
| #28 | Limit #27 to English, Human and Publication year (1995 to Current) | 1923 |

1. **PsycINFO (Ovid Interface, 1987 to May Week 4, 2016)**

| **Search Number** | **Search String** | **Results** |
| --- | --- | --- |
| #1 | exp Meta Analysis/ | 3534 |
| #2 | exp Methodology/ | 79331 |
| #3 | Treatment Effectiveness Evaluation/ | 17621 |
| #4 | exp Experimentation/ | 58524 |
| #5 | Evidence Based Practice/ | 13410 |
| #6 | exp Experimental Design/ | 41170 |
| #7 | Clinical Trial/ | 9520 |
| #8 | or/#1 – #7 | 183003 |
| #9 | ((approach or approaches or standard*1 or system*1 or instrument*1 or framework*1 or level*1 or hierarchy or hierarchies or method* or profile*1 or guideline*1 or guide*1) adj3 evidence).ti. | 1415 |
| #10 | (quality or confidence or certainty or strength).tw. | 252400 |
| #11 | ((rate or rates or rating or grade or grades or grading or measure or measuring or score or scoring or assess or assessing or evaluate or evaluating or tool*1 or checklist*1 or system*1 or instrument*1) adj3 ((level*1 or hierarchy or hierarchies) adj3 evidence)).tw. | 101 |
| #12 | #9 and #10 | 230 |
| #13 | #10 and #11 | 47 |
| #14 | ((grade or grades or grading or rate or rates or rating or evaluate or evaluating or evaluation or assess*) adj3 evidence).ti. | 666 |
| #15 | ((grade or grades or grading or rate or rates or rating or evaluate or evaluating or assess or assessing or quality or strength) adj3 recommendation*1).ti. | 44 |
| #16 | ((framework*1 or system*1 or instrument*1) adj2 evidence).ti. | 172 |
| #17 | #12 or #13 or #14 or #15 or #16 | 1123 |
| #18 | ((grade or grades or grading or rate or rates or rating or assess* or evaluate or evaluating or appraisal or appraising) adj2 (quality or strength) adj5 (evidence or recommendation*1)).ti. | 16 |
| #19 | ((method or methods or methodology or approach or approaches) adj3 (assess or assessing or assessment or evaluate or evaluating) adj3 (recommendation*1 or evidence)).ti. | 19 |
| #20 | (good adj3 practi#e*1 adj3 recommendation*1).tw. | 72 |
| #21 | (#8 and #17) or #18 or #19 or #20 | 515 |
| #22 | Limit #21 to English, Human and Publication year (1995 to Current) | 479 |

1. **EMBASE (Ovid Interface, 1988 to 2016 Week 22)**

| **Search Number** | **Search String** | **Results** |
| --- | --- | --- |
| #1 | exp Research/ | 582597 |
| #2 | exp Meta Analysis/ | 109010 |
| #3 | exp Systematic Review/ | 106868 |
| #4 | Epidemiology/ | 106658 |
| #5 | Randomized Controlled Trial/ | 387363 |
| #6 | Evidence Based Practice/ | 43451 |
| #7 | Practice Guideline/ | 275322 |
| #8 | Intervention/ | 83 |
| #9 | Clinical Trial/ | 812713 |
| #10 | or/#1–#9 | 1955325 |
| #11 | ((approach or approaches or standard*1 or system*1 or instrument*1 or framework*1 or level*1 or hierarchy or hierarchies or method* or profile*1 or guideline*1 or guide*1) adj3 evidence).ti. | 5922 |
| #12 | (quality or confidence or certainty or strength).tw. | 1427287 |
| #13 | ((rate or rates or rating or grade or grades or grading or measure or measuring or score or scoring or assess or assessing or evaluate or evaluating or tool*1 or checklist*1 or system*1 or instrument*1) adj3 ((level*1 or hierarchy or hierarchies) adj3 evidence)).tw. | 1036 |
| #14 | #11 and #12 | 1192 |
| #15 | #12 and #13 | 467 |
| #16 | ((grade or grades or grading or rate or rates or rating or evaluate or evaluating or evaluation or assess*) adj3 evidence).ti. | 1916 |
| #17 | ((grade or grades or grading or rate or rates or rating or evaluate or evaluating or assess or assessing or quality or strength) adj3 recommendation*1).ti. | 493 |
| #18 | ((framework*1 or system*1 or instrument*1) adj2 evidence).ti. | 443 |
| #19 | #14 or #15 or #16 or #17 or #18 | 4297 |
| #20 | ((grade or grades or grading or rate or rates or rating or assess* or evaluate or evaluating or appraisal or appraising) adj2 (quality or strength) adj5 (evidence or recommendation*1)).ti. | 129 |
| #21 | ((method or methods or methodology or approach or approaches) adj3 (assess or assessing or assessment or evaluate or evaluating) adj3 (recommendation*1 or evidence)).ti. | 57 |
| #22 | (good adj3 practi#e*1 adj3 recommendation*1).tw. | 401 |
| #23 | (#10 and #19) or #20 or #21 or #22 | 2776 |
| #24 | Limit #23 to English, Human and Publication year (1995 to Current) | 1996 |

1. **Cochrane Methodology Register and Cochrane Groups – Cochrane Library (1995 to 2016)**

| **Search Number** | **Search String** | **Results** |
| --- | --- | --- |
| #1 | Title, Abstract, Keywords: (((approach or approaches or standard* or system* or instrument* or framework* or level* or hierarchy or hierarchies or method* or profile* or guideline* or guide*) near/3 evidence) and (quality or confidence or certainty or strength)) | 395 |
| #2 | Title, Abstract, Keywords:  (good near/3 practice* near/3 recommendation*) | 2 |
| #3 | Record Title:  ((grade or grades or grading or rate or rates or rating or evaluate or evaluating or assess or assessing or quality or strength) near/3 (evidence or recommendation*)) | 98 |
| #4 | Record Title:  ((grade or grading or rate or rating or assess or assessing or evaluate or evaluating or appraisal or appraising) near/2 (quality or strength) near/5 evidence) | 22 |
| #5 | Record Title:  ((framework* or system* or instrument*) near/2 evidence) | 25 |
| #6 | #1 or #2 or #3 or #4 or #5 | 457 |

1. **Scopus Social Sciences (1960 to Present)**

| **Search Number** | **Search String** | **Results** |
| --- | --- | --- |
| #1 | TITLE((approach or approaches or standard* or system* or instrument* or framework* or level* or hierarchy or hierarchies or method* or profile* or guideline* or guide*) W/3 evidence) | 1578 |
| #2 | TITLE-ABS-KEY(quality or confidence or certainty or strength) | 228403 |
| #3 | #1 and #2 | 245 |
| #4 | TITLE-ABS-KEY(good W/3 practice* W/3 recommendation*) | 303 |
| #5 | TITLE((framework* or systems* or instrument*) W/2 evidence) | 357 |
| #6 | TITLE((grade or grades or grading or rate or rates or rating or evaluate or evaluating or assess or assessing) W/3 evidence) | 431 |
| #7 | TITLE((grade or grades or grading or rate or rates or rating or evaluate or evaluating or assess or assessing or quality or strength) W/3 recommendation*) | 52 |
| #8 | TITLE((grade or grading or rate or rating or assess* or evaluate or evaluating or appraisal or appraising) W/2 (quality or strength) W/5 evidence) | 22 |
| #9 | TITLE((method or methods or methodology or approach or approaches) W/3 (assess or assessing or assessment or evaluate or evaluating) W/3 (evidence or recommendation*)) | 14 |
| #10 | #3 or #4 or #5 or #6 or #7 or #8 or #9 | 1315 |
| #11 | Limit 8 to English, Publication Year (1995 to 2016) | 1205 |

1. **Social Science Citation Index (SSCI); Web of Science^TM^ Core Collection (1956 to 2016)**

| **Search Number** | **Search String** | **Results** |
| --- | --- | --- |
| #1 | TI=((approach or approaches or standard* or system* or instrument* or framework* or level* or hierarchy or hierarchies or method* or profile* or guideline* or guide*) near/3 evidence) | 4072 |
| #2 | TS=(quality or confidence or certainty or strength) | 383959 |
| #3 | #1 and #2 | 699 |
| #4 | TS=(good near/3 practice* near/3 recommendation*) | 415 |
| #5 | TI=((framework* or system* or instrument*) near/2 evidence) | 836 |
| #6 | TI=((grade or grades or grading or rate or rates or rating or evaluate or evaluating or assess or assessing) near/3 evidence) | 1266 |
| #7 | TI=((grade or grades or grading or rate or rates or rating or evaluate or evaluating or assess or assessing or quality or strength) near/3 recommendation*) | 96 |
| #8 | TI=((grade or grading or rate or rating or assess* or evaluate or evaluating or appraisal or appraising) near/2 (quality or strength) near/5 evidence) | 47 |
| #9 | TI=((methods or method or methodology or approach or approaches) near/3 (assess or evaluate or assessment or assessing or evaluating) near/3 (evidence or recommendation*)) | 46 |
| #10 | #3 or #4 or #5 or #6 or #7 or #8 or #9 | 3154 |
| #11 | Limit 8 to English and Publication Year (1995 to 2016) | 2857 |

1. **Applied Social Sciences Index and Abstracts (1987-2016)**

| **Search Number** | **Search String** | **Results** |
| --- | --- | --- |
| #1 | ti((approach or approaches or standard* or level* or hierarchy or hierarchies or method* or framework* or system* or instrument* or profile* or guideline* or guide*) near/3 evidence) | 604 |
| #2 | ab(quality or confidence or certainty or strength) | 59539 |
| #3 | 1 and 2 | 129 |
| #4 | ti(good near/3 practice* near/3 recommendation*) OR ab(good near/3 practice* near/3 recommendation*) | 147 |
| #5 | ti((framework* or system* or instrument*) near/2 evidence) | 131 |
| #6 | ti((grade or grades or grading or rate or rates or rating or evaluate or evaluating or assess or assessing) near/3 evidence) | 124 |
| #7 | ti((grade or grades or grading or rate or rates or rating or evaluate or evaluating or assess or assessing or quality or strength) near/3 recommendation*) | 23 |
| #8 | ti((grade or grading or rate or rating or assess* or evaluate or evaluating or appraisal or appraising) near/2 (quality or strength) near/5 evidence) | 14 |
| #9 | ti((methods or method or methodology or approach or approaches) near/3 (assess or evaluate or assessment or assessing or evaluating) near/3 (evidence or recommendation*)) | 5 |
| #10 | #3 or #4 or #5 or #6 or #7 or #8 or #9 | 518 |
|  | Limit #10 to Publication Year (1995 to 2016) | 491 |

1. **SCIE Social Care Online**

| **Search Number** | **Search String** | **Results** |
| --- | --- | --- |
| #1 | Title:  framework or system or instrument or grade or rate or assess or evaluate or method or methodology or approach or level or hierarchy or appraisal or guideline or guide | 19073 |
| #2 | Abstract: evidence | 12957 |
| #3 | Abstract:  quality or strength | 13041 |
| #4 | #1 and #2 and #3 | 351 |
| #5 | All fields: “strength of evidence” or “strength of recommendation” or “quality of evidence” or “quality of recommendation” | 131 |
| #6 | #4 or #5 | 452 |

**Appendix 2. Searches on the Websites of Key Stakeholder Organisations**

| **Organisation Website** | **Search** |
| --- | --- |
| [Agency for Healthcare Research and Quality](https://www.effectivehealthcare.ahrq.gov/index.cfm/search-for-guides-reviews-and-reports/) | **Date:** 11.05.2016  **Search performed:** *evidence grading* (in the Search for Research Summaries, Reviews, and Reports. Effective Health Care Program)  **Number of records retrieved and screened:** 237 |
| [Appraisal of Guidelines for Research and Evaluation (AGREE)](http://www.agreetrust.org/) | **Date:** 24.05.2016  **Search performed:** hand searched the Resource Center and Research Projects sections of the website  **Number of records retrieved and screened:** 8 |
| [Campbell Collaboration](http://www.campbellcollaboration.org/) | **Date:** 23.05.2016  **Search performed:** hand searched the Methods Group, Campbell Methods Series, Campbell Policies and Guidelines and Resource Center sections of the website  **Number of records retrieved and screened:** 7 |
| [Canadian Task Force on Preventive Health Care (CTFPHC)](http://canadiantaskforce.ca/) | **Date:** 23.05.2016  **Search performed:** hand searched the Methods section of the website  **Number of records retrieved and screened:** 1 |
| [Centre for the Development and Evaluation of Complex Interventions for Public Health Improvement (DECIPHer)](http://decipher.uk.net/) | **Date:** 25.05.2016  **Search performed:** *evidence or grading or rating or assessing or quality or strength* (searched in the Title of the website Publications)  **Number of records retrieved and screened:** 37 |
| [Centre for Diet and Activity Research (CEDAR)](http://www.cedar.iph.cam.ac.uk/) | **Date:** 25.05.2016  **Search performed:** *evidence grading or evidence rating or strength of evidence or quality of evidence or quality assessment* (searched for Keywords in Titles and Abstracts of website Publications); also hand searched the Resources section of the website  **Number of records retrieved and screened:** 8 |
| [Centre for Evidence-Based Crime Policy](http://cebcp.org/) | **Date:** 24.05.2016  **Search performed:** hand searched the Research Programs section of the website  **Number of records retrieved and screened:** 7 |
| [Centre for Evidence-Based Intervention (CEBI)](http://www.spi.ox.ac.uk/research/details/grade-extension-for-complex-social-inter.html) | **Date:** 23.05.2016  **Search performed:** hand searched the Methodology section of the website  **Number of records retrieved and screened:** 1 |
| [Centre for Evidence-Based Medicine (CEBM)](http://www.cebm.net/) | **Date:** 23.05.2016  **Search performed:** hand searched the EBM Resources section of the website  **Number of records retrieved and screened:** 4 |
| [Centre for Reviews and Dissemination (CRD)](http://www.york.ac.uk/crd/) | **Date:** 24.05.2016  **Search performed:** hand searched the Our Guidance section of the website and the CRD Database: Title: *rating or grading or (assessing adj5 quality) or (strength and evidence)*  **Number of records retrieved and screened:** 34 |
| [Centre for Translational Research in Public Health (Fuse)](http://www.fuse.ac.uk/) | **Date:** 25.05.2016  **Search performed:** hand searched the Research Section of the website  **Number of records retrieved and screened:** 13 |
| [Centre of Excellence for Public Health Northern Ireland](http://www.qub.ac.uk/research-centres/CentreofExcellenceforPublicHealthNorthernIreland//) | **Date:** 25.05.2016  **Search performed:** hand searched the Research and Publications sections of the website  **Number of records retrieved and screened:** 8 |
| [Cochrane Collaboration](http://uk.cochrane.org/) | **Date:** 27.05.2016  **Search performed:** hand searched Cochrane Methods Group, Cochrane and EPOC Resources for Authors section of the website  **Number of records retrieved and screened:** 51 |
| [Critical Appraisal Skills Programme (CASP)](http://www.casp-uk.net/) | **Date:** 24.05.2016  **Search performed:** hand searched the CASP Tools and Checklists Section of the website  **Number of records retrieved and screened:** 40 |
| [Developing and Evaluating Communication Strategies to Support Informed Decisions and Practice Based on Evidence (DECIDE)](http://www.decide-collaboration.eu/) | **Date:** 25.05.2016  **Search performed:** hand searched the Publications and Other Dissemination Activities section of the website  **Number of records retrieved and screened:** 6 |
| [Department for Education](https://www.gov.uk/government/organisations/department-for-education) | **Date:** 20.05.2016  **Search performed:** *strength of evidence or evidence grading or evidence rating or quality of evidence*  **Number of records retrieved and screened:** 676 |
| [Department of Health](https://www.gov.uk/government/organisations/department-of-health) | **Date:** 20.05.2016  **Search performed:** *strength of evidence or evidence grading or evidence rating or quality of evidence*  **Number of records retrieved and screened:** 374 |
| [Department for International Development (DFID)](https://www.gov.uk/government/organisations/department-for-international-development) | **Date:** 20.05.2016  **Search performed:** *strength of evidence or evidence grading or evidence rating or quality of evidence*  **Number of records retrieved and screened:** 184 |
| [ESRC UK Centre for Evidence Based Policy and Practice](http://www.researchcatalogue.esrc.ac.uk/grants/H141251005/read/outputs) | **Date:** 07.06.2016  **Search performed:** hand searched the outputs of the project  **Number of records retrieved and screened:** 32 |
| [EQUATOR Network](http://www.equator-network.org/) | **Date:** 25.05.2016  **Search performed:** Reporting Guidelines for Systematic Reviews  **Number of records retrieved and screened:** 24 |
| [European Centre for Disease Prevention and Control](http://ecdc.europa.eu/en/Pages/home.aspx) | **Date:** 25.05.2016  **Search performed:** *evidence grading or evidence rating or strength of evidence* (searched conducted in Publications, All sites, News and Events sections of the website)  **Number of records retrieved and screened:** 156 |
| [Evidence for Policy and Practice Information and Co-ordinating Centre (EPPI-Centre)](http://eppi.ioe.ac.uk/cms/Default.aspx?tabid=1919#appraising) | **Date:** 16.05.2016  **Search performed:** hand searched publications on systematic review/evidence synthesis methodology: appraising and synthesising evidence  **Number of records retrieved and screened:** 19 |
| [GRADE Working Group](http://www.gradeworkinggroup.org/) | **Date:** 26.05.2016  **Search performed:** hand searched the Publication section of the website  **Number of records retrieved and screened:** 19 |
| [Guidelines International Network (G-I-N)](http://www.g-i-n.net/) | **Date:** 26.05.2016  **Search performed:** hand searched the Working Groups and Resources sections of the website  **Number of records retrieved and screened:** 37 |
| [International Initiative for Impact Evaluation (3ie)](http://www.3ieimpact.org/en/evidence/impact-evaluations/) | **Date:** 23.05.2016  **Search performed:** hand searched the Resources and Systematic Reviews sections of the website  **Number of records retrieved and screened:** 1 |
| [Joanna Briggs Institute (JBI)](http://joannabriggs.org/) | **Date:** 23.05.2016  **Search performed:** hand searched the website  **Number of records retrieved and screened:** 6 |
| [Ministry of Justice](http://www.justice.gov.uk/) | **Date:** 20.05.2016  **Search performed:** *strength of evidence or evidence grading or evidence rating or quality of evidence*  **Number of records retrieved and screened:** 119 |
| [National Foundation for Educational Research (NFER)](http://www.nfer.ac.uk/about-nfer/) | **Date:** 20.05.2016  **Search performed:** *assessing evidence or strength of evidence or evidence grading or evidence rating* (search in the publications)  **Number of records retrieved and screened:** 88 |
| [National Guideline Clearinghouse](http://www.guideline.gov/index.aspx) | **Date:** 31.05.2016  **Search performed:** hand searched the website (Guideline Matrix and Guideline Resources sections)  **Number of records retrieved and screened:** 2 |
| [National Health and Medical Research Council (NHMRC)](https://www.nhmrc.gov.au/guidelines-publications/information-guideline-developers/resources-guideline-developers) | **Date:** 12.05.2016  **Search performed:** hand searched the resources for guideline developers section of the website  **Number of records retrieved and screened:** 15 |
| [National Institute for Health and Care Excellence](https://www.nice.org.uk/) | **Date:** 11.05.2016  **Search performed:** hand searched the process and methods guides sections of the website:  *evidence grading*  **Number of records retrieved and screened:** 42 |
| [NHS Health Development Agency](http://www.webarchive.org.uk/wayback/archive/20140616143352/http://nice.org.uk/aboutnice/whoweare/aboutthehda/hdapublications/hda_publications.jsp) | **Date:** 12.05.2016  **Search performed:**  searched all the titles of HDA publications  **Number of records retrieved and screened:** 606 |
| [Norwegian Institute of Public Health](http://www.fhi.no/) | **Date:** 25.05.2016  **Search performed:** hand searched Research Projects section of the website  **Number of records retrieved and screened:** 27 |
| [Public Health Agency of Canada](http://www.phac-aspc.gc.ca/about_apropos/index-eng.php) | **Date:** 07.06.2016  **Search performed:** quality of strength (in the title of documents)  **Number of records retrieved and screened:** 15 |
| [Scottish Intercollegiate Guidelines Network](http://www.sign.ac.uk/) | **Date:** 23.05.2016  **Search performed:** hand searched the Methodology section of the website  **Number of records retrieved and screened:** 4 |
| [Social Care Institute for Excellence](http://www.scie.org.uk/) | **Date:** 23.05.2016  **Search performed:** hand searched the Research & Knowledge section of the website: Knowledge review, Guide and Research resource  **Number of records retrieved and screened:** 86 |
| [Specialist Unit for Review Evidence (SURE)](http://www.cardiff.ac.uk/insrv/libraries/sure/index.html) | **Date:** 25.05.2016  **Search performed:** hand searched the Projects, Publications, Resources for Systematic Reviewers and Critical Appraisal Checklists sections of the website  **Number of records retrieved and screened:** 151 |
| [The Public Health Agency of Sweden](https://www.folkhalsomyndigheten.se/the-public-health-agency-of-sweden/) | **Date:** 13.10.2016  **Search performed:** contacted the website for an English version guidance  **Number of records retrieved and screened: 0** |
| [The National Board of Health and Welfare (Socialstyrelsen)’s MetodGuiden: Sweden](http://www.socialstyrelsen.se/evidensbaseradpraktik) | **Date:** 13.10.2016  **Search performed:** contacted the website for an English version guidance  **Number of records retrieved and screened: 1** |
| [US Community Preventive Services Task Force](https://www.thecommunityguide.org/publications) | **Date:** 12.05.2016  **Search performed:** hand searched the “Methods” of the Publication section of the website  **Number of records retrieved and screened:** 3 |
| [USAID Development Experience Clearinghouse](https://dec.usaid.gov/dec/content/evaluations.aspx) | **Date:** 27.05.2016  **Search performed: evidence** *grading or evidence rating or strength or quality of evidence* (Titles of the Documents)  **Number of records retrieved and screened:** 78 |
| [Vanderbilt University Evidence-Based Practice Center](http://medicineandpublichealth.vanderbilt.edu/epc/index.php) | **Date:** 27.05.2016  **Search performed:** *grading or rating or strength* (Titles and Abstracts of the Projects and Publications)  **Number of records retrieved and screened:** 13 |
| [WHO evidence-informed policy-making: Health Evidence Network (HEN)](http://www.euro.who.int/en/data-and-evidence/evidence-informed-policy-making/health-evidence-network-hen) | **Date:** 31.05.2016  **Search performed:** “evidence grading” or “evidence rating” or “quality of evidence” or “strength of evidence” (searched HEN Sources of Evidence Database)  **Number of records retrieved and screened:** 44 |
| [WHO evidence-informed policy-making: Evidence-informed Policy Network (EVIPNet)](http://www.euro.who.int/en/data-and-evidence/evidence-informed-policy-making/evidence-informed-policy-network-evipnet) | **Date:** 31.05.2016  **Search performed:** hand searched the website (including the Resources for Evidence-Based Policy section of EVIPNet Global website)  **Number of records retrieved and screened:** 151 |
| [WHO Guidelines](http://www.who.int/publications/guidelines/en/) | **Date:** 31.05.2016  **Search performed:** searched the text of all the guidelines for “GRADE” or “strength” or “quality”  **Number of records retrieved and screened:** 154 |
| [World Bank Impact Evaluation: Open Knowledge Repository](https://openknowledge.worldbank.org/) | **Date:** 26.05.2016  **Search performed:** evidence or grading or rating or quality or strength or confidence (search limited to Titles only)  **Number of records retrieved and screened:** 223 |

**US Clearinghouses**

| Best Evidence Encyclopedia | <http://www.bestevidence.org/index.cfm> |
| --- | --- |
| Best Practices Registry for Suicide Prevention | <http://www.sprc.org/strategic-planning/finding-programs-practices> |
| California Evidence-Based Clearinghouse for Child Welfare (CEBC) | [http://www.cebc4cw.org](http://www.cebc4cw.org/) |
| California Healthy Kids Resource Center | <http://www.californiahealthykids.org/index> |
| Center for Knowledge Translation for Disability and Rehabilitation Research | [http://ktdrr.org](http://ktdrr.org/) |
| CrimeSolutions.gov | [http://CrimeSolutions.gov](http://crimesolutions.gov/) |
| Evidence-Based Practices for Substance Use | <http://lib.adai.washington.edu/ebpsearch.htm> |
| FindYouthInfo.gov | <http://www.findyouthinfo.gov/> |
| Home Visiting Evidence on Effectiveness | [http://homvee.acf.hhs.gov](http://homvee.acf.hhs.gov/) |
| My Brother's Keeper | <http://mbk.ed.gov/> |
| National Guideline Clearinghouse | See above |
| National Registry of Evidence-based Programs and Practices (NREPP) | <http://nrepp.samhsa.gov/01_landing.aspx> |
| Office of Adolescent Health: Teen Pregnancy Prevention Evidence-Based Programs | <http://www.hhs.gov/ash/oah/> |
| Office of Juvenile Justice and Delinquency Prevention (OJJDP). Model Programs Guide | [http://www.ojjdp.gov](http://www.ojjdp.gov/) |
| OJJDP's Strategic Planning Tool | <https://www.nationalgangcenter.gov/SPT/> |
| Promise Neighborhoods Research Consortium | <http://promiseneighborhoods.org/index.html> |
| Research-tested Intervention Programs | <http://rtips.cancer.gov/rtips/index.do> |
| Strengthening Families Evidence Reviews | [http://familyreview.acf.hhs.gov](http://familyreview.acf.hhs.gov/) |
| The Community Guide | See above |
| The Clearinghouse for Labor Evaluation and Research (CLEAR) | [http://clear.dol.gov](http://clear.dol.gov/) |
| United States Interagency Council on Homelessness' Solutions Database | <https://www.usich.gov/solutions> |
| Washington State Institute for Public Policy | [http://www.wsipp.wa.gov](http://www.wsipp.wa.gov/) |
| What Works Clearinghouse (WWC, Department of Education) | <http://ies.ed.gov/ncee/wwc/> |
| What Works in Reentry Clearinghouse (WWR) | [https://whatworks.csgjusticecenter.org](https://whatworks.csgjusticecenter.org/) |

**UK What Works Network**

| Affiliate: Public Policy Institute for Wales | [http://ppiw.org.uk](http://ppiw.org.uk/) |
| --- | --- |
| Affiliate: What Works Scotland | [http://whatworksscotland.ac.uk](http://whatworksscotland.ac.uk/) |
| Centre for Ageing Better | <http://www.centreforageingbetter.com/> |
| Early Intervention Foundation | <http://guidebook.eif.org.uk/> |
| Education Endowment Foundation | <http://educationendowmentfoundation.org.uk/toolkit/> |
| National Institute for Health and Care Excellence | See above |
| What Works Centre for Crime Reduction | <http://www.college.police.uk/en/20018.htm> |
| What Works Centre for Local Economic Growth | <http://whatworksgrowth.org/> |
| What Works Centre for Wellbeing | [https://whatworkswellbeing.org](https://whatworkswellbeing.org/) |

**Other Agencies**

| Centre for Excellence and Outcomes in Children and Young People's Services | <http://www.c4eo.org.uk/home.aspx> |
| --- | --- |
| Child family Communities Australia | <https://aifs.gov.au/cfca/> |
| Commissioning Toolkit – Parenting Programmes | <https://www.education.gov.uk/commissioning-toolkit/Programme/Index> |
| Investing In Children | <http://investinginchildren.eu/> |
| Nesta Standards of Evidence | <http://www.nesta.org.uk> |
| Project Oracle - Children & Youth Evidence Hub: UK | <http://project-oracle.com/> |
| The Edna McConnell Clark Foundation | <http://www.emcf.org/our-strategy/selection-process/evidence/> |

**Appendix 3. Data Extraction Form**

An electronic data extraction form has been developed based on the following items. Excel 2016 with macros will be used for data extraction.

**Descriptive Information:**

- Date of extraction
- Extractor initials
- Document ID
- Document authors
- Document title
- Publication year
- Title of the system
- Publication sources
  - Journal
  - Agency website
- Name of the source
- Type of system
  - Generic
  - Specific
- Specification (if specific, e.g., specific discipline/field, intervention, outcome, etc.)
- Practice domains
  - Clinical medicine
  - Criminology
  - Education
  - International development
  - Nursing
  - Psychology
  - Public health
  - Social work
  - Other (specify)
- Purpose of the system
  - Evidence synthesis
  - Guideline development
  - Both

**Document Eligibility:**

- Document eligibility
  - Eligible
  - Not eligible
  - Uncertain
- Reason (if ineligible)

**Document Content:**

- Definition of the *quality of a body of evidence* (i.e., how does the system suggest to define the construct of the quality of a body of evidence)
- Evidence synthesis approach (i.e., how does the system suggest to synthesise evidence on intervention effectiveness, if at all)
- Domains for rating the quality of a body of evidence
  - Name of the domain
  - Definition of the domain
  - Criteria used to assess the domain
  - Definition of the criteria used to assess the domain
  - Procedure for rating the domain
- Other domains described in the system (beyond effectiveness evidence)
  - Name of the domain
  - Definition of the domain
  - Criteria used to assess the domain
  - Definition of the criteria used to assess the domain
  - Procedure for rating the domain
- Categories of ratings
  - Rating category (e.g., “grade 1” or “high”)
  - Definition of the category

**System Development:**

- Did the developers describe whether they identified previous relevant domains of evidence and/or identified key limitations of these? Describe how?
- Did the developers report any participants involved in the development of the system? Describe how?
- Did the developers report obtaining any funding for the development of the system? Describe how?
- Did the developers report conducting a Delphi exercise? Describe how?
- Did the developers report conducting an expert meeting? Describe how?
- Did the authors describe the process of reaching consensus on the system? Describe how? E.g., discuss rationale for including certain standards in the system.

**System Write-up & Dissemination:**

- Did the developers discuss how the document describing the system was written? Describe how? E.g., Pilot testing, drafting and revising within the team.
- Did the developers provide an explanatory document and instructions for using the system? Describe how?
- Did the developers describe how they planned to deal or dealt with criticism and feedback for the system? Describe how?
- Is the system placed and available in an open-access website? Describe how?
- Did the developers report any processes for seeking adherence to the system? Describe how?
- Has the system been translated into other languages?

1. The search was limited to titles, because text word search retrieved 52385 publications

   [↑](#footnote-ref-1)
