# Supplementary material for: Rating the quality of a body of evidence on the effectiveness of health and social interventions: A systematic review and mapping of evidence domains
Source: Res Synth Methods. 2018 Mar 2;9(2):224–42. doi: 10.1002/jrsm.1290 (PMC6001464; doi:10.1002/jrsm.1290)
Supplement: Supplementary file 1 — File S1. Review protocol File S2. Specification of the evidence domains in the included evidence rating systems [file JRSM-9-224-s001.zip › Supplementary File 2.docx]

**Supplementary File 2. Specification of the evidence domains in the included evidence rating systems**

| **Baral et al. (2012)**  **The Highest Attainable Standard of Evidence (HASTE)** | | |
| --- | --- | --- |
| ***Domain*** | ***Definition*** | ***Criteria for the domain (converted into signaling questions)*** |
| Efficacy data | N/D | N/D |
| **Berkman et al. (2013)**  **Agency for Healthcare Research and Quality (AHRQ)** | | |
| ***Domain*** | ***Definition*** | ***Criteria for the domain (converted into signaling questions)*** |
| Design (D) | N/D | - D1: What was the study design? - Randomised controlled trial (high quality) - Observational study (low quality) |
| Study Limitations in RCTs/CCTs (SLR) | It refers to the judgment that the findings from included studies of a treatment (or treatment comparison) for a given outcome are adequately protected against bias (i.e., have good internal validity), based on the design and conduct of those studies. | - SLR1: Was the allocation sequence generated adequately (e.g., random number table, computer- generated randomisation)? (i.e., no potential for selection bias) - SLR2: Was the allocation of treatment adequately concealed (e.g., pharmacy-controlled randomisation or use of sequentially numbered sealed envelopes)? (i.e., no potential for selection bias) - SLR3: Were participants analysed within the groups they were originally assigned to? (i.e., no potential for selection bias) - SLR4: Does the design or analysis control account for important confounding and modifying variables through matching, stratification, multivariable analysis, or other approaches? (i.e., no potential for selection bias) - SLR5: Did researchers rule out any impact from a concurrent intervention or an unintended exposure bias that might bias results? (i.e., no potential for performance bias) - SLR6: Did the study maintain fidelity to the intervention protocol? (i.e., no potential for performance bias) - SLR7: If attrition (overall or differential nonresponse, dropout, loss to follow-up, or exclusion of participants) was a concern, were missing data handled appropriately (e.g., intention-to-treat analysis and imputation)? (i.e., no potential for attrition bias) - SLR8: In prospective studies, was the length of follow-up different between the groups, or in case-control studies, was the time period between the intervention/exposure and outcome the same for cases and controls? (i.e., no potential for detection bias) - SL9: Were the outcome assessors blinded to the intervention or exposure status of participants? (i.e., no potential for detection bias) |
|  |  | - SLR10: Were interventions/exposures assessed/defined using valid and reliable measures, implemented consistently across all study participants? (i.e., no potential for detection bias) - SLR11: Were outcomes assessed/defined using valid and reliable measures, implemented consistently across all study participants? (i.e., no potential for detection bias) - SLR12: Were confounding variables assessed using valid and reliable measures, implemented consistently across all study participants? (for CCTs only; i.e., no potential for detection bias) - SLR13: Were the potential outcomes pre-specified by the researchers? Are all pre-specified outcomes reported? (i.e., no potential for reporting bias) |
| Study Limitations in Case-Control Studies (SLCC) | The same as for RCTs/CCTs | - SLCC1: Were cases and controls selected appropriately (e.g. appropriate diagnostic criteria or definitions, equal application of exclusion criteria to case and controls, sampling not influenced by exposure status)? (i.e., no potential for selection bias) - SLCC2: Does the design or analysis control account for important confounding and modifying variables through matching, stratification, multivariable analysis, or other approaches? (i.e., no potential for selection bias) - SLCC3: Did researchers rule out any impact from a concurrent intervention or an unintended exposure bias that might bias results? (i.e., no potential for performance bias) - SLCC4: Did the study maintain fidelity to the intervention protocol? (i.e., no potential for performance bias) - SLCC5: If attrition (overall or differential nonresponse, dropout, loss to follow-up, or exclusion of participants) was a concern, were missing data handled appropriately (e.g., intention-to-treat analysis and imputation)? (i.e., no potential for attrition bias) - SLCC6: In prospective studies, was the length of follow-up different between the groups, or in case-control studies, was the time period between the intervention/exposure and outcome the same for cases and controls? (i.e., no potential for detection bias) - SLCC7: Were the outcome assessors blinded to the intervention or exposure status of participants? (i.e., no potential for detection bias) - SLCC8: Were interventions/exposures assessed/defined using valid and reliable measures, implemented consistently across all study participants? (i.e., no potential for detection bias) - SLCC9: Were outcomes assessed/defined using valid and reliable measures, implemented consistently across all study participants? (i.e., no potential for detection bias) - SLCC10: Were confounding variables assessed using valid and reliable measures, implemented consistently across all study participants? (i.e., no potential for detection bias) - SLCC11: Were the potential outcomes pre-specified by the researchers? Are all pre-specified outcomes reported? (i.e., no potential for reporting bias) |
| Study Limitations in Cross-Sectional Studies (SLCS) | The same as for RCTs/CCTs | - SLCS1: Did the study apply inclusion/exclusion criteria uniformly to all comparison groups? (i.e., no potential for selection bias) - SLCS2: Does the design or analysis control account for important confounding and modifying variables through matching, stratification, multivariable analysis, or other approaches? (i.e., no potential for selection bias) - SLCS3: Did researchers rule out any impact from a concurrent intervention or an unintended exposure bias that might bias results? (i.e., no potential for performance bias) - SLCS4: If attrition (overall or differential nonresponse, dropout, loss to follow-up, or exclusion of participants) was a concern, were missing data handled appropriately (e.g., intention-to-treat analysis and imputation)? (i.e., no potential for attrition bias) - SLCS5: Were outcome assessors blinded to the intervention or exposure status of participants? - SLCS6: Were interventions/exposures assessed/defined using valid and reliable measures, implemented consistently across all study participants? (i.e., no potential for detection bias) - SLCS7: Were outcomes assessed/defined using valid and reliable measures, implemented consistently across all study participants? (i.e., no potential for detection bias) - SLCS8: Were confounding variables assessed using valid and reliable measures, implemented consistently across all study participants? (i.e., no potential for detection bias) - SLCS9: Were the potential outcomes pre-specified by the researchers? Are all pre-specified outcomes reported? (i.e., no potential for reporting bias) |
| Study Limitation in Case Series (SLSeries) | The same as for RCTs/CCTs | - SLSeries1: Does the design or analysis control account for important confounding and modifying variables through matching, stratification, multivariable analysis, or other approaches? (i.e., no potential for selection bias) - SLSeries2: Did researchers rule out any impact from a concurrent intervention or an unintended exposure bias that might bias results? (i.e., no potential for performance bias) - SLSeries3: Did the study maintain fidelity to the intervention protocol? (i.e., no potential for performance bias) - SLSeries4: If attrition (overall or differential nonresponse, dropout, loss to follow-up, or exclusion of participants) was a concern, were missing data handled appropriately (e.g., intention-to-treat analysis and imputation)? (i.e., no potential for attrition bias) - SLSeries5: Were outcome assessors blinded to the intervention or exposure status of participants? - SLSeries6: Were interventions/exposures assessed/defined using valid and reliable measures, implemented consistently across all study participants? (i.e., no potential for detection bias) - SLSeries7: Were outcomes assessed/defined using valid and reliable measures, implemented consistently across all study participants? (i.e., no potential for detection bias) - SLSeries8: Were confounding variables assessed using valid and reliable measures, implemented consistently across all study participants? (i.e., no potential for detection bias) - SLSeries9: Were the potential outcomes pre-specified by the researchers? Are all pre-specified outcomes reported? (i.e., no potential for reporting bias) |
| Consistency (C) | Consistency refers to the degree of similarity in the direction of effects or the degree of similarity in the effect sizes (magnitudes of effect) across individual studies within an evidence base. EPCs may choose which of these two notions of consistency (direction or magnitude) they are scoring; they should be explicit about this choice. | - C1: What was the consistency in direction of effect estimates in relation to the line that distinguishes superiority from inferiority (or minimally important difference for non-inferiority or equivalence)? - C1.1: To what extent did confidence intervals overlap? - C2: What was the consistency in the magnitude of effect? - C2.1: Was the statistical test for heterogeneity (Cochran’s Q test) significant? - C2.2: Was the magnitude of statistical heterogeneity (as measured by I^2^) large? - C2.3: Did point estimates vary widely?   Note: The consistency of a single-study evidence base is judged as unknown. |
| Directness (D) | Directness of evidence expresses how closely available evidence measures an outcome of interest. Assessing directness has two parts: directness of outcomes and directness of comparisons. Applicability of evidence (external validity) is considered explicitly but separately from strength of evidence. | - D1: Was the included outcome an intermediate or a proxy of an ultimate health outcomes? - D2: Did investigators use proxy respondents to stand in for certain kinds of patients or subjects in measuring the outcome of interest? - D3: Were the conclusions based on direct (head-to-head) comparisons? |
| Precision (P) | Precision is the degree of certainty surrounding an estimate of effect with respect to an outcome. It is based on the potential for random error evaluated through the sufficiency of sample size and, in the case of dichotomous outcomes, the number of events. | - P1: What was the width of the confidence interval around the pooled effect estimate? - P2: Was the optimal information size criterion met? - P3: What was the potential for random error in individual studies (specifically when a quantitative synthesis is not possible? |
| Reporting Bias (RB) | Reporting bias occurs when authors, journals, or both decide to publish or report research findings based on their direction or magnitude of effect. There are three main types of reporting bias that authors or journals can introduce:   - Publication bias - Selective outcome reporting bias - Selective analysis reporting | - RB1: Did the authors conduct a quantitative assessment of the “missingness” of outcome data from small studies? - RB1.1 Tests of funnel plot asymmetry; trim and fill method; selection modeling - RB2: Did the authors conduct a qualitative assessment of the risk of reporting bias (considers 7 factors below)? - RB2.1: Estimated number of studies affected by reporting biases - RB2.2: Total sample size affected by reporting biases - RB2.3: Total number of studies in evidence base - RB2.4: Total number of participants in evidence base - RB2.5: Consistency of effect estimates across contributing studies - RB2.6: Study limitation of the evidence base - RB2.7: Comprehensiveness of study retrieval and identification |
| Large Magnitude of Effect (ME) | Strength of association refers to the likelihood that the observed effect is large enough that it cannot have occurred solely as a result of bias from potential confound factors. This additional domain should be considered when the effect size is particularly large. | - ME1: Was there large magnitude of effect? |
| Dose-Response Relationship (DR) | This association, either across or within studies, refers to a pattern of a larger effect with greater exposure (dose, duration, adherence). This domain should be considered when studies in the evidence base have noted levels of exposure. | - DR1: Was there a dose-response relationship between the intervention and the outcome? |
| Plausible confounding (PC) | Occasionally, in an observational study, plausible confounding would work in the direction opposite that of the observed. This additional domain should be considered when plausible confounding exists that would decrease the observed effect. | - PC1: Would plausible confounding decrease the observed effect? |
| **Briss et al. (2000)**  **The Guide to Community Preventive Services** | | |
| ***Domain*** | ***Definition*** | ***Criteria for the domain (converted into signaling questions)*** |
| Design Suitability (D) | Suitability of study design is characterised based on several characteristics that help to protect against a variety of potential threats to validity. | - D1: Is the study design suitable to protect against a variety of potential threats to validity? - Concurrent comparison (greatest suitability) - Comparison, but not concurrent (moderate suitability) - Single group (least suitability) |
| Study Execution (SE) | Reviewers assess quality of study execution by considering six categories of threats to validity (see the criteria below). | **Description**   - SE1: Was the study population (i.e. the intervention and comparison population) well described? - SE2: Was the intervention well described?   **Sampling**   - SE3: Did the authors specify (i.e. describe characteristics and size of) the sampling frame or universe of selection for the study population? - SE4: Did the authors specify the screening criteria for study eligibility (if applicable)? - SE5: Was the population that served as the unit of analysis the entire eligible population or a probability sample at the point of observation? - SE6: Are there other selection bias issues not identified above?   **Measurement**   - SE7: Was there an attempt to measure exposure to the intervention? - SE8: Were the exposure variables valid measures of the intervention under study? - SE8.1: Clear definition of the exposure variable - SE8.2: Measurement of exposure in different ways - SE8.3: Citations of discussion as to why the use of these measures is valid - SE9: Were the exposure variables reliable (consistent and reproducible) measures of the intervention under study? - SE9.1: Measures of internal consistency - SE9.2: Measurement of exposure in different ways - SE9.3: Inter-rater reliability checks - SE9.4: Citations or discussion as to why the use of these measures is reliable - SE10: Were the outcome and other independent (or predictor) variables valid measures of the outcome of interest? - SE10.1: Clear definition of the outcome variable - SE10.2: Measurement of the outcome in different ways - SE10.3: Citations or discussion as to why the use of these measures is valid - SE11: Were the outcome and other independent (or predictor) variables reliable (consistent and reproducible) measures of the outcome of interest? - SE11.1: Measures of internal validity - SE11.2: Measurement of the outcome in different ways - SE11.3: Considered consistency of coding, scoring or categorization between observers or between different outcome measures - SE11.4: Considered how setting and sampling of study population might affect reliability   **Analysis**   - SE12: Did the authors conduct appropriate analysis - SE12.1: Conducting statistical testing? - SE12.2 Reporting which statistical tests were used? - SE12.3: Controlling for design effects in the statistical model? - SE12.4: Controlling for repeated measures in the analysis, for study designs in which the same population was followed with repeated measurements over time? - SE12.5: Accounting for different levels of exposure in segments of the study population in the analysis? - SE12.6: If the authors analyzed group-level and individual-level covariates in the same statistical model, was the model designed to handle multi-level data? - SE13: Were there other problems with data analysis that limit interpretation of the results of the study?   **Interpretation of results**   - SE14: Did at least 80% of enrolled participants (i.e. intervention and comparison groups) complete the study? - SE15: Did the authors assess whether the units of analyses were comparable prior to exposure to the intervention? - SE16: Considering the study design, were appropriate methods for controlling confounding variables and limiting potential biases used? - SE17: Did the authors identify and discuss potential biases or unmeasured/contextual confounder that may account for or influence the observed results and explicitly state how they assessed these potential confounders and biases?   **Other**   - SE18: Are there other issues that limit your ability to interpret the results of the study that were not identified handled in one of the other categories? |
| Number of Studies (N) | N/D | - N1: How many studies contribute to the evidence base? |
| Consistency (C) | N/D | - C1: Were findings generally consistent in direction and size? |
| Magnitude of Effect (ME) | Sufficient and large effect sizes are defined on a case-by-case basis and are based on Task Force | - ME1: Was there large effect size? |
| **Bruce et al. (2014)**  **Grading of Evidence for Public Health Interventions (GEPHI)** | | |
| ***Domain*** | ***Definition*** | ***Criteria for the domain (converted into signaling questions)*** |
| Design (D) | N/D | - D1: What was the study design? - Randomised controlled trial (high quality) - Quasi-experimental [with controls] and before and after [uncontrolled] studies: this would be the case so long as the evidence from these studies can clearly be shown to be stronger in terms of minimising selection bias and confounding than other observational designs (moderate quality) - Observational study (low quality) |
| Analogy (A) | Coherent evidence on the effect of similar environmental health interventions or exposures that operate through the same or a similar mechanism. | - A1: Is there coherence evidence on the effect of similar environmental health intervention or exposures that operate through the same or a similar mechanism? |
| Consistency (C) | Consistent evidence is found across a large number of settings, geographical locations and/or over time, and across diverse epidemiological study designs and/or gathered by different researchers. | - C1: Is consistent evidence found across a large number of settings, geographical locations and/or over time, and across diverse epidemiological study designs and/or gathered by different researchers? |
| Coherence (C) | Coherence of evidence contributing to the causal chain. | - C1: Is there coherence in the body of evidence contributing to the causal chain? |
| **Clark et al. (2009)**  **Let Evidence Guide Every New Decision (LEGEND)** | | |
| ***Domain*** | ***Definition*** | ***Criteria for the domain (converted into signaling questions)*** |
| Study Quality for RCTs (SQR) | The aggregate quality ratings for individual studies (including their design) | ***Validity: are the results of the RCT valid or credible?***   - SQR1: Were the patients randomly assigned to treatment and control groups? - SQR2: Was that randomisation conducted appropriately? - SQR2.1: Was the randomization concealed from those responsible for recruiting subjects? - SQR2.2: Were patients, parents, clinicians, and analysts masked to which treatment was being received? - SQR3: Were the groups similar at the start of the trial, with respect to known prognostic factors? - SQR4: Aside from the experimental treatment, were the groups treated equally? - SQR5: Were all patients who entered the trial accounted for at its conclusion? - SQR5.1: Was there a low rate of attrition? (Note: if greater than 20% lost to follow up, bias may be of greater concern) - SQR6: Were patients accounted for (and analyzed) in the groups to which they were randomized (i.e. ITT analysis)? - SQR7: Was the study long enough to fully study effects of the intervention? - SQR8: Were instruments used to measure the outcomes valid and reliable? - SQR9: Was there freedom from conflict of interest?   ***Reliability: are these valid study results important?***   - SQR10: Did the study have a sufficiently large sample size? - SQR10.1: Was there a power analysis? - SQR10.2: Did the sample size achieve or exceed that resulting from the power analysis? - SQR10.3: Did each subgroup also have sufficient sample size (e.g. at least 6 to 12 participants)? - SQR11: What were the main results of the RCT? - SQR11.1: What was the effect size? (How large was the treatment effect?) - SQR11.2: What were the measures of statistical uncertainty (e.g. precision)? - SQR12: Were the results statistically significant? - SQR13: Were the results clinically significant? - SQR13.1: If potential confounders were identified, were they discussed in relationship to the results? - SQR14: Were adverse events assessed?   ***Applicability: can I apply these valid, important study results to treating my patients?***   - SQR15: Can the results be applied to my population of interest? - SQR15.1: Is the treatment feasible in my care setting? - SQR15.2: Do the patient outcomes apply to my population or question of interest? - SQR15.3: Are the likely benefits worth the potential harm and costs? - SQR15.4: Were the patients in this study similar to my population of interest? - SQR16: Are my patient’s and family’s values and preferences satisfied by the treatment and its consequences?   SQR17: Would you include this study/article in development of a care recommendation? |
| Study Quality for Systematic Reviews and Meta-Analyses (SQS) | The same as for RCTs | ***Validity: are the results of the systematic review/meta-analyses valid or credible?***   - SQS1: Did the overview address a focused clinical question? - SQS2: Was the search for relevant studies detailed and exhaustive? - SQS2.1: Was it unlikely that important, relevant studies were missed? - SQS3: Did the systematic review use RCTs? - SQS3.1: Were the criteria used to select articles for inclusion appropriate? - SQS3.2: Was the assignment of patients to treatments randomized? - SQS4: Were the included studies appraised and assigned a high level of quality? - SQS5: Were the methods consistent from study to study? - SQS5.1: Were populations among the included studies comparable and appropriate? - SQS5.2: Were the outcomes, interventions, and exposures measured in the same way? - SQS6: Was there freedom from conflict of interest? - SQS6.1: Sponsor/Funding Agency or Investigators?   ***Reliability: are these valid study results important?***   - SQS7: What were the main results of the systematic review/meta-analysis? - SQS7.1: What was the effect size? (how large was the treatment effect?) - SQS7.2: What were the measures of statistical uncertainty (e.g. precision)? - SQS8: Were the results statistically significant? - SQS9: Were the results clinically significant? - SQS9.1: If potential confounders were identified, were they discussed in relationship to the results? - SQS10: Were adverse events discussed?   ***Applicability: can I apply these valid, important study results to treating my patients?***   - SQS11: Can the results be applied to my population of interest? - SQS11.1: Is the treatment feasible in my care setting? - SQS11.2: Do the patient outcomes apply to my population or question of interest? - SQS11.3: Are the likely benefits worth the potential harm and costs? - SQS11.4: Are the patients in this study similar to my population of interest? - SQS12: Are my patient’s and family’s values and preferences satisfied by the treatment and its consequences? - SQS13: Would you include this study/ article in development of a care recommendation? |
| Study Quality for Longitudinal Studies  (e.g. time series) (SQL) | Same as for RCTs | ***Validity: are the results of the longitudinal study valid or credible?***   - SQL1: Were the study methods appropriate for the question? - SQL1.1: Were the study methods clearly described (e.g. setting, sample population)? - SQL1.2: Were data collected at more than one point in time (i.e. before/after, pretest/posttest, time series)? - SQL2: Were instruments used to measure the outcomes valid and reliable? - SQL2.1: Were the instruments tested to be reliable? - SQL3: Were all appropriate variables (e.g. potential confounders, exposures, predictors) and interventions clearly described? - SQL4: Were all appropriate outcomes clearly described? - SQL5: Was there freedom from conflict of interest? - SQS5.1: Sponsor/Funding Agency or Investigators.   ***Reliability: are these valid study results important?***   - SQL6: Were the statistical analysis methods appropriate? - SQL6.1: Were the statistical analysis methods clearly described? - SQL7: Did the study have a sufficiently large sample size? - SQL7.1: Was a power analysis described? - SQL7.2: Did the sample size achieve or exceed that resulting from the power analysis? - SQL7.3: Did each subgroup also have sufficient sample size (e.g., at least 6-12 participants)? - SQL8: What were the main results of the study? - SQL8.1: What was the effect size? - SQL8.2: What were the measures of statistical uncertainty (e.g. precision? - SQL9: Were the results statistically significant? - SQL10: Were the results clinically significant? - SQL10.1: If potential confounders were identified, were they discussed in relationship to the results? - SQL11: Were any adverse events assessed?   ***Applicability: can I apply these valid, important study results to treating my patients?***   - SQL12: Can the results be applied to my population of interest? - SQL12.1: Is the treatment feasible in my care setting? - SQL12.2: Do the patient outcomes apply to my population or question of interest? - SQL12.3: Are the likely benefits worth the potential harm and costs? - SQL12.4: Were the patients in this study similar to my population of interest? - SQS12: Are my patient’s and family’s values and preferences satisfied by the treatment and its consequences? - SQL13: Would you include this study/article in development of a care recommendation? |
| Study Quality for Cohort Studies (SQC) | The same as for RCTs | ***Validity: are the results of the cohort study valid or credible?***   - SQC1: Were the study methods appropriate for the question? - SQC1.1: Were the study methods clearly described (e.g. setting, sample population)? - SQC1.2: Were the instruments clearly described? - SQC1.3: Were the interventions clearly described? - SQC2: Were the participants recruited prospectively with a comparison group? - SQC3: Were instruments used to measure the outcomes valid and reliable? - SQC3.1: Were the instruments tested to be valid and reliable? - SQC4: Were all appropriate variables (e.g. potential confounders, exposures, predictors) and interventions clearly described? - SQC5: Were all appropriate outcomes clearly described? - SQC6: Was the follow-up process described and complete? - SQC6.1: Was the follow-up long enough to fully study the effects of the intervention? - SQC6.2: Was there a low rate of attrition? (20%) - SQC7: Was there freedom from conflict of interest? - SQC7.1: Sponsor/Funding Agency or Investigators.   ***Reliability: are these valid study results important?***   - SQC8: Were the statistical analysis methods appropriate? - SQC8.1: Were the statistical analysis methods clearly described? - SQC9: Did the study have a sufficiently large sample size? - SQC9.1: Was a power analysis described? - SQC9.2: Did the sample size achieve or exceed that resulting from the power analysis? - SQC9.3: Did each subgroup also have sufficient sample size (e.g., at least 6-12 participants)? - SQC10: What were the main results of the study? - SQC10.1: What was the effect size? - SQC10.2: What were the measures of statistical uncertainty (e.g. precision)? - SQC11: Were the results statistically significant? - SQC12: Were the results clinically significant? - SQC13.1: If potential confounders were identified, were they discussed in relationship to the results? - SQC14: Were any adverse events assessed?   ***Applicability: can I apply these valid, important study results to treating my patients?***   - SQC15: Can the results be applied to my population of interest? - SQC15.1: Is the treatment feasible in my care setting? - SQC15.2: Do the patient outcomes apply to my population or question of interest? - SQC15.3: Are the likely benefits worth the potential harm and costs? - SQC15.4: Were the patients in this study similar to my population of interest? - SQC16: Are my patient’s and family’s values and preferences satisfied by the treatment and its consequences? - SQC17: Would you include this study/article in development of a care recommendation? |
| Study Quality for Case-Control Studies (SQCC) | The same as for RCTs | ***Validity: are the results of the case-control study valid or credible?***   - SQCC1: Were the study methods appropriate for the question? - SQCC1.1: Were the study methods clearly described (e.g. setting, sample population)? - SQCC1.2: Were cases and controls matched appropriately for confounders or comorbidities? - SQCC1.3: Were appropriate numbers of control participants matched to the case participants? - SQCC2: Were instruments used to measure the outcomes valid and reliable? - SQCC2.1: Were the instruments tested to be reliable? - SQCC3: Were all appropriate variables (e.g. potential confounders, exposures, predictors) and interventions clearly described? - SQCC4: Were all appropriate outcomes clearly described? - SQCC5: Were all participants accounted for at the conclusion of the study? - SQCC5.1: Were missing data explained? - SQCC6: Was there freedom from conflict of interest? - SQCC6.1: Sponsor/Funding Agency or Investigators.   ***Reliability: are these valid study results important?***   - SQCC7: Were the statistical analysis methods appropriate? - SQCC7.1: Were the statistical analysis methods clearly described? - SQCC8: Did the study have a sufficiently large sample size? - SQCC8.1: Was a power analysis described? - SQCC8.2: Did the sample size achieve or exceed that resulting from the power analysis? - SQCC8.3: Did each subgroup also have sufficient sample size (e.g., at least 6-12 participants)? - SQCC9: What were the main results of the study? - SQCC9.1: What was the effect size? - SQCC9.2: What were the measures of statistical uncertainty (e.g. precision)? - SQCC10: Were the results statistically significant? - SQCC11: Were the results clinically significant? - SQCC11.1: If potential confounders were identified, were they discussed in relationship to the results? - SQCC12: Were any adverse events assessed?   ***Applicability: can I apply these valid, important study results to treating my patients?***   - SQCC13: Can the results be applied to my population of interest? - SQCC13.1: Is the treatment feasible in my care setting? - SQCC13.2: Do the patient outcomes apply to my population or question of interest? - SQCC13.3: Are the likely benefits worth the potential harm and costs? - SQCC13.4: Were the patients in this study similar to my population of interest? - SQCC14: Are my patient’s and family’s values and preferences satisfied by the treatment and its consequences? - SQCC15: Would you include this study/article in development of a care recommendation? |
| Study Quality for Cross-Sectional Studies (SQCS) | The same as for RCTs | ***Validity: are the results of the case-control study valid or credible?***   - SQCS1: Were the study methods appropriate for the question? - SQCS1.1: Were the study methods clearly described (e.g. setting, sample population)? - SQCS1.2: Were the instruments clearly described? - SQCS1.3: Were the data collected at one point in time? - SQCS2: Were instruments used to measure the outcomes valid and reliable? - SQCS2.1: Were the instruments tested to be valid and reliable? - SQCS3: Were all appropriate variables (e.g. potential confounders, exposures, predictors) and interventions clearly described? - SQCS4: Were all appropriate outcomes clearly described? - SQCS5: Were all participants accounted for at the conclusion of the study? - SQCS5.1: Were withdrawals from the study explained? - SCCS5.2: Was the rate of attrition acceptable? - SQCS6: Was there freedom from conflict of interest? - SQCS6.1: Sponsor/Funding Agency or Investigators.   ***Reliability: are these valid study results important?***   - SQCS7: Were the statistical analysis methods appropriate? - SQCS7.1: Were the statistical analysis methods clearly described? - SQCS8: Did the study have a sufficiently large sample size? - SQCS8.1: Was a power analysis described? - SQCS8.2: Did the sample size achieve or exceed that resulting from the power analysis? - SQCS8.3: Did each subgroup also have sufficient sample size (e.g., at least 6-12 participants)? - SQCS9: What were the main results of the study? - SQCS9.1: What was the effect size? - SQCS9.2: What were the measures of statistical uncertainty (e.g. precision)? - SQCS10: Were the results statistically significant? - SQCS11: Were the results clinically significant? - SQCS11.1: If potential confounders were identified, were they discussed in relationship to the results? - SQCS12: Were any adverse events assessed?   ***Applicability: can I apply these valid, important study results to treating my patients?***   - SQCS13: Can the results be applied to my population of interest? - SQCS13.1: Is the treatment feasible in my care setting? - SQCS13.2: Do the patient outcomes apply to my population or question of interest? - SQCS13.3: Are the likely benefits worth the potential harm and costs? - SQCS13.4: Were the patients in this study similar to my population of interest? - SQCS14: Are my patient’s and family’s values and preferences satisfied by the treatment and its consequences? - SQCS15: Would you include this study/article in development of a care recommendation? |
| Study Quality for Case Series (SQSeries) |  | ***Basic elements of a case report***   - SQSeries1: Does the case report fit into one of the categories expected? - SQSeries1.1: New associations or variations in disease processes - SQSeries1.2: Innovative approaches/interventions for treatment in disease processes - SQSeries1.3: Findings that shed light on the possible pathogenesis of a disease or an adverse event - SQSeries1.4: Presentations, diagnoses, or management of new and emerging diseases - SQSeries1.5: Unreported or unusual side effects or adverse interactions involving medications or treatment - SQSeries1.6: Unexpected or unusual presentations of a disease - SQSeries1.7: Unexpected association between diseases and symptoms - SQSeries1.8: Unexpected event in the course of observing or treating a patient - SQSeries1.9: Does not fit one of the categories expected for case reports - SQSeries2: Does the case report include a background of the issue on which the case report focuses? - SQSeries3: Does the case report include an up-to-date review of the previous cases in the field? - SQSeries4: Does the case report include details relevant to the case? - SQSeries4.1: A description of the patient’s demographic information - SQSeries4.2: Any relevant medical history of the patient or their family - SQSeries4.3: The patient’s signs and symptoms - SQSeries4.4: Any tests that were carried out - SQSeries4.5: A description of pertinent details (e.g. disorder, medication, interventions/treatments, adverse events) - SQSeries4.6: The outcome of the case - SQSeries5: Is the importance of the case explained? - SQSeries5.1: What can be learned from the case report? - SQSeries5.2: How will the case report advance our clinical knowledge? - SQSeries5.3: Other? - SQSeries6: Was there freedom from conflict of interest? - SQSeries6.1: Sponsor/Funding Agency or Investigators/Authors   ***Applicability: can I apply these valid, important study results to treating my patients?***   - SQSeries7: Can the results be applied to my population of interest? - SQSeries7.1: Is the treatment feasible in my care setting? - SQSeries7.2: Was the patient (or were the patients) in this report similar to my population of interest? - SQSeries7.3: Do the patient outcomes apply to my population or question of interest? - SQSeries8: Are my patient’s and family’s values and preferences satisfied by the treatment and its consequences? - SQSeries9: Would you include this report in development of a care recommendation? |
| Consistency (C) | The extent to which similar findings are reported using similar and different study designs. | - C1: To what extent are similar findings reported using similar and different study designs? |
| Number of Studies (N) | N/D | - N1: How many studies contribute to the evidence base? |
| Magnitude of Effect (ME) | N/D | ME1: Was there large effect size? |
| **Department for International Development (DFID, 2014)**  **How to Note: assessing the strength of evidence** | | |
| ***Domain*** | ***Definition*** | ***Criteria for the domain (converted into signaling questions)*** |
| Quality (Q) | The (technical) quality of the studies constituting the body of evidence (or the degree to which risk of bias has been addressed). | Conceptual framing   - Q1: Does the study acknowledge existing research? - Q2: Does the study construct a conceptual framework? - Q3: Does the study pose a research question or outline a hypothesis?   Transparency   - Q4: Does the study present or link to the raw data it analyses? - Q5: What is the geography/context in which the study was conducted? - Q6: Does the study declare sources of support/funding?   Appropriateness   - Q7: Does the study identify a research design? - Q8: Does the study identify a research method? - Q9: Does the study demonstrate why the chosen design and method are well suited to the research question?   Cultural sensitivity   - Q10: Does the study explicitly consider any context‐specific cultural factors that may bias the analysis/findings?   Validity   - Q11: To what extent is the study internally valid? - Q12: To what extent is the study externally valid? - Q13: To what extent is the study ecologically valid?   Reliability   - Q14: To what extent are the measures used in the study stable? - Q15: To what extent are the measures used in the study internally reliable? - Q16: To what extent are the findings likely to be sensitive/changeable depending on the analytical technique used?   Cogency   - Q17: Does the author ‘signpost’ the reader throughout? - Q18: To what extent does the author consider the study’s limitations and/or alternative interpretations of the analysis?   Q19: Are the conclusions clearly based on the study’s results? |
| Size (S) | Size of the body of evidence | S1: How many studies contribute to the evidence base? |
| Consistency (C) | A range of studies pointing to identical or similar conclusions | C1: Do a range of studies point to identical or similar conclusions? |
| Context (C) | Context of the body of evidence | C1: What is the context of the body of evidence? |
| **Ebell et al. (2004)**  **Strength of Recommendation Taxonomy (SORT)** | | |
| ***Domain*** | ***Definition*** | ***Criteria for the domain (converted into signaling questions)*** |
| Study Quality | The aggregate quality ratings for individual studies (including their design) | N/D |
| Consistency (C) | N/D | - C1: To what extent to the studies point to identical, or similar conclusions? |
| **Gough et al. (2007)**  **Weight of Evidence: a framework for the appraisal of the quality and relevance of evidence** | | |
| ***Domain*** | ***Definition*** | ***Criteria for the domain (converted into signaling questions)*** |
| Study Execution | This is a generic and thus non review specific judgement about the coherence and integrity of the evidence in its own terms. | N/D |
| Relevance of Design (D) | This is a review specific judgement about the appropriateness of that form of evidence for answering the review question, that is the fitness for purpose of that form of evidence. | - D1: Is the study design appropriate for answering the review question? |
| Relevance of Context/Focus of Evidence (R) | This is a review specific judgement about the relevance of the focus of the evidence for the review question. | - R1: Is the focus of evidence relevant for answering the review question (e.g. sample, analysis, context of the evidence, ethics of the research behind the evidence)? |
| **Hillier et al. (2011)**  **FORM: an Australian method for formulating and grading recommendations in evidence-based guidelines** | | |
| ***Domain*** | ***Definition*** | ***Criteria for the domain (converted into signaling questions)*** |
| Evidence base  (Design; D) | The level of evidence indicates the study design used by the investigators to assess the effectiveness of an intervention. The level assigned to a study reflects the degree to which bias has been eliminated by the study design. Level of evidence reflects the best study types for the specific type of question. | - D1: What level of study type (design) was used to answer the question? - Systematic review of RCTs (level I) - RCT (level II) - Pseudorandomised controlled trial (level III-1) - Comparative study with concurrent controls (level III-2) - Comparative study without concurrent controls (level III-3) - Case series with either post-test or pre-test/post-test outcomes (level-IV) |
| Evidence base  (Study Quality; SQ) | The quality of the evidence refers to the methods used by the investigators during the study to minimise bias and control confounding within a study type (i.e. how well the investigators conducted the study). Study quality relates to an assessment of the risk of bias inherent in the conduct, design and reporting of results in the included studies. | Method of treatment assignment   - SQ1: Did the study describe a correct, blinded randomisation method and document the group similarity?   Control of selection bias after treatment assignment   - SQ2: Did the study adhere to intention-to-treat analysis and full follow-up of the sample?   Blinding   - SQR3: Did the study report blinding of outcome assessors and patient and care givers?   Outcome assessment (if blinding was not possible)   - SQ4: Did the study use standardised measures of assessment? |
| Evidence base  (Quantity; Q) | Quantity of evidence reflects the number of the studies that have been included as the evidence base for each guideline. | - Q1: How many studies contribute to the evidence base? |
| Consistency (C) | The consistency component of the “body of evidence” assesses the extent to which the findings are consistent across the included studies (including across a range of study populations and study designs). This allows users to assess whether the results are likely to be replicable or only likely to occur under certain conditions. | - C1: To what extent are the findings consistent across the included studies? - C2: Was the magnitude of statistical heterogeneity (as measured by e.g. I^2^) large? - C3: Was the direction of effect across multiple studies consistent? |
| Clinical Impact (CI) | Clinical impact is a measure of the likely benefit that application of the guideline would have across the target population, and involves a clinical judgement. | - CI1: Was evidence base relevant to the clinical question? - CI2: Was the treatment effect statistically significant (low *P*-value)? - CI3: Was the size (magnitude) of the treatment effect clinically important (did the confidence interval include a clinically important effect)? - CI4: What is the relevance of the effect to patients, compared to other management options? - CI5: What is the duration of therapy required to achieve the effect? - CI6: What is the balance of risks of benefits to the patient group, including potential harms? |
| Applicability (A) | Applicability addresses whether the evidence base is relevant to the Australian health care system generally, or to more local settings for specific recommendations (such as rural areas or cities). | - A1: Is the evidence base applicable in terms of organizational factors (e.g. availability of trained stud, clinic time, specialized equipment, tests or other resources)? - A2: Is the evidence base applicable in terms of cultural factors (e.g. attitudes to health issues, including those that may effect compliance with the recommendation)? |
| Generalisability (G) | The assessment of generalisability involves determining how precisely the available body of evidence answers the clinical question that was asked. | - G1: How well do the participants of the included studies match the patient population being targeted by the guideline? - G2: How well do the clinical settings of the included studies match the settings where the recommendation will be implemented? - G3: How well do the stages of disease considered in the included studies match those being targeted by the guideline? - G4: How well does the duration of illness considered in the included studies match that being targeted by the guideline? - G5: How well does the prevalence of the disease considered in the population of included studies match that being targeted by the guideline? |
| **Joanna Brigs Institute (2014)**  **Levels of evidence and grades of recommendations** | | |
| ***Domain*** | ***Definition*** | ***Criteria for the domain (converted into signaling questions)*** |
| Design (D) | N/D | - D1: What level of study type (design) was in the evidence base? - Experimental Designs (level 1) - Quasi-experimental Designs (level 2) - Observational – analytic designs (level 3) - Observational – descriptive studies (level 2) - Expert opinion and bench research (level 1) |
| **Johnson et al. (2015)**  **Introducing EMMIE: an evidence rating scale to encourage mixed-method crime prevention synthesis** | | |
| ***Domain*** | ***Definition*** | ***Criteria for the domain (converted into signaling questions)*** |
| Methodological adequacy of evidence in terms of estimating effect sizes (MA) | Assessing the level of bias in the estimates of mean effect sizes | - MA1: Did the review use transparent well-designed search strategy? - MA2: Did the authors assess the influence of study design by means of moderator analysis? - MA3: Did the authors pay sufficient attention to the validity of the constructs, with only comparable outcomes combined, and/or exploration of the implications of combining outcome constructs? - MA4: Did the authors assess the influence of unanticipated outcomes or spin-offs on the size of the effect? - MA5: Did the authors assess publication bias? - MA6: Did the authors consider inter-coder reliability? - MA7: Did the authors consider the influence of statistical outliers? |
| **National Institute for Health and Care Excellence (NICE, 2012)**  **Methods for the development of NICE public health guidance** | | |
| ***Domain*** | ***Definition*** | ***Criteria for the domain (converted into signaling questions)*** |
| Study Quality (SQ) | Internal ( that is, to check if potential sources of bias have been minimised and to determine if its conclusions are open to any degree of doubt) ; External (i.e. the extent to which the findings for the study participants are generalisable to the whole 'source population' (that is, the population they were chosen from). | Population   - SQ1: Is the source population or source area well described? Was the country, setting, location, population demographics, etc. adequately described? - SQ2: Is the eligible population or area representative of the source population or area? Was the recruitment of individuals, clusters or areas well defined? Was the eligible population representative of the source? Were important groups under-represented - SQ3: Do the selected participants or areas represent the eligible population or area? Was the method of selection of participants from the eligible population well described What % of selected individuals or clusters agreed to participate? Were there any sources of bias? Were the inclusion or exclusion criteria explicit and appropriate?   Method of allocation   - SQ4: How was selection bias minimised? Was allocation to exposure and comparison randomised? Was it truly random, or pseudo-randomised (e.g. consecutive admissions)? If not randomised, was significant confounding likely or not? If a cross-over, was order of intervention randomised? - SQ5: Were interventions (and comparisons) well described and appropriate? Were interventions and comparisons described in sufficient detail (i.e. enough for study to be replicated)? Was comparisons appropriate (e.g. usual practice rather than no intervention)? - SQ6: Was the allocation concealed? Could the person(s) determining allocation of participants or clusters to intervention or comparison groups have influenced the allocation? - SQ7: Were participants or investigators blind to exposure and comparison? Were participants and investigators – those delivering or assessing the intervention kept blind to intervention allocation? - SQ8: Was the exposure to the intervention and comparison adequate? Is reduced exposure to intervention or control related to the intervention (e.g. adverse effects leading to reduced compliance) or fidelity of implementation (e.g. reduced adherence to protocol)? Was lack of exposure sufficient to cause important bias? - SQ9: Was contamination acceptably low? Did any in the comparison group receive the intervention or vice versa? If so, was it sufficient to cause important bias? If a cross-over trial, was there a sufficient wash-out period between interventions? - SQ10: Were other interventions similar in both groups? Did either group receive additional interventions or have services provided in a different manner? Were the groups treated equally by researchers or other professionals? Was this sufficient to cause important bias? - SQ11: Were all participants accounted for at study conclusion? Were those lost-to-follow-up (i.e. dropped or lost pre-, during or post- intervention) acceptably low (i.e. typically <20%)? Did the proportion dropped differ by group? - SQ12: Did the setting reflect usual UK practice? Did the setting in which the intervention or comparison was delivered differ significantly from usual practice in the UK? - SQ13: Did the intervention or control comparison reflect usual UK practice? Did the intervention or comparison differ significantly from usual practice in the UK?   Outcomes   - SQ14: Were outcome measures reliable? Were outcome measures subjective or objective? How reliable were outcome measure? Was there any indication that measures had been validated? - SQ15: Were all outcome measurements complete? Were all or most study participants who met the defined study outcome definitions likely to have been identified? - SQ16: Were all important outcomes assessed? Were all important benefits and harms assessed? Was it possible to determine the overall balance of benefits and harms of the intervention versus comparison? - SQ17: Were outcomes relevant? Where surrogate outcome measures were used, did they measure what they set out to measure? - SQ18: Were there similar follow-up times in exposure and comparison groups? - SQ19: Was follow-up time meaningful? Was follow-up long enough to assess long-term benefits or harms? Was it too long, e.g. participants lost to follow-up?   Analyses   - SQ20: Were exposure and comparison groups similar at baseline? If not, were these adjusted? Were there any differences between groups in important confounders at baseline? If so, were these adjusted for in the analyses (e.g. multivariate analyses or stratification). Were there likely to be any residual differences of relevance? - SQ21: Was intention to treat (ITT) analysis conducted? Were all participants (including those that dropped out or did not fully complete the intervention course) analysed in the groups (i.e. intervention or comparison) to which they were originally allocated? - SQ22: Was the study sufficiently powered to detect an intervention effect (if one exists)? Is a power calculation presented? If not, what is the expected effect size? Is the sample size adequate? - SQ23: Were the estimates of effect size given or calculable? Were effect estimates (e.g. relative risks, absolute risks) given or possible to calculate? - SQ24: Were the analytical methods appropriate? Were important differences in follow-up time and likely confounders adjusted for? If a cluster design, were analyses of sample size (and power), and effect size performed on clusters (and not individuals)? Were subgroup analyses pre-specified? - SQ25: Was the precision of intervention effects given or calculable? Were they meaningful? Were confidence intervals or *P-*values for effect estimates given or possible to calculate? Were CI's wide or were they sufficiently precise to aid decision-making? If precision is lacking, is this because the study is under-powered?   Summary   - SQ26: Are the study results internally valid (i.e. unbiased)? How well did the study minimise sources of bias (i.e. adjusting for potential confounders)? Were there significant flaws in the study design?   SQ27: Are the findings generalisable to the source population (i.e. externally valid)? Are there sufficient details given about the study to determine if the findings are generalisable to the source population? |
| Quantity (Q) | N/D | - Q1: How many studies contribute to the evidence base? |
| Consistency (C) | N/D | - C1: How consistent were the findings? |
| **Sawaya et al. (2007)**  **U.S. Preventive Services Task Force (USPSTF)** | | |
| ***Domain*** | ***Definition*** | ***Criteria for the domain (converted into signaling questions)*** |
| Design (D) | N/D | - D1: Do the studies have the appropriate research design to answer the key questions (i.e., different linkages in the causal pathway/analytic framework)? |
| Study Quality for Systematic Reviews (SQS) | To what extent are the existing studies (comprising the body of evidence) of high quality? (i.e. what is the internal validity)? | - SQS1: Was the used sources/search strategy comprehensive? - SQS2: Did the review appraise included studies? - SQS3: Were conclusions valid? - SQS4: Is review relevant and recent? |
| Study Quality for Randomized Controlled Trials (RCTs; SQR) | The same as for Systematic Reviews | - SQR1: Did the study adequately randomise participants, including allocation concealment, and were confounders equally distributed among groups? - SQR2: Did the study maintain comparable groups throughout (attrition, crossover, adherence, contamination? - SQR3: Was there important differential loss to follow-up or overall high loss to follow-up? - SQR4: Did the study employ reliable and valid measures (including masking of outcome assessment)? - SQR5: Were interventions clearly described? - SQR6: Were all important outcomes considered? - SQR7: Did study adjust for potential confounders for cohort studies, or employ intention-to-treat analysis? |
| Study Quality for Case-Control Studies (SQCC) | The same as for Systematic Reviews | - SQCC1: Were cases accurately ascertained? - SQCC2: Did the study employ nonbiased selection of cases/controls with exclusion criteria applied equally to both? - SQCC3: What was the response rate in the sample? - SQCC4: Were diagnostic testing procedures applied equally to each group? - SQCC5: Did study pay appropriate attention to potential confounding variables? |
| Quantity (Magnitude of Effect; Q/ME) | N/D | - Q1/ME1: How many studies have been conducted that address the key question(s) (i.e. different linkages in the causal pathway/analytic framework)? How large are the studies? (i.e., what is the precision of the evidence?) |
| Generalisability (G) | N/D | - G1: To what extent are the results of the studies generalisable to the general U.S. primary care population and situation? (i.e., what is the external validity?) |
| Consistency (C) | N/D | - C1: How consistent are the results of the studies? |
| Dose-Response (DR) |  | - DR1: Are there additional factors that assist us in drawing conclusions (e.g., presence or absence of dose–response effects, fit within a biologic model)? |
| Other | N/D | N/D |
| **Tang et al. (2007)**  **Grading of evidence of the effectiveness of health promotion interventions** | | |
| ***Domain*** | ***Definition*** | ***Criteria for the domain (converted into signaling questions)*** |
| Association (A) | To grade evidence, it is imperative to find out whether or not an intervention works, e.g. high and presumably statistically significant association between the intervention and the outcome factors, such as indicated by a relative risk and its confidence interval. | - A1: Was there large magnitude of effect? - RR of 2 of more - A2: Was the effect statistically significant? |
| Repeatability (R) | It is then important to find out whether the intervention is widely repeatable, e.g. in different countries and settings. This reflects the consistency of the findings in different studies. | - R1: Was the intervention widely repeatable in different countries and settings? |
| How it works (H) | It is also important to find out how it works - the theoretical basis for making an association between the intervention and the outcome factors. If the theoretical basis is not known, the strength of evidence will be less convincing. | - H1: Does the intervention have a known theoretical basis? |
| **Treadwell et al. (2006)**  **A system for rating the stability and strength of medical evidence** | | |
| ***Domain*** | ***Definition*** | ***Criteria for the domain (converted into signaling questions)*** |
| Study Quality (SQ) | Quality of evidence for a specific outcome. Although quality evaluation can be performed with a checklist or scale, any reasonable method for separating the evidence base into different categories of quality will suffice. | N/D |
| Quantity (Q) | Number of studies for each outcome. | - Q1: Were there at least 3 studies for the outcome of interest? - Q2: Did a certain percentage of studies (e.g. 80% or more) have calculable effect sizes (that can be determined without imputation)? |
| Informativeness (I) | This use of "informativeness" accounts for the statistical power of the evidence base | - I1: Is the treatment beneficial? - I2: Is the treatment effect clinically important (i.e., the lower 95% confidence interval around the meta-analytic summary statistic is greater than the effect size deemed clinically important [decided a priori])? |
| Homogeneity (H) | Statistical homogeneity testing (using fixed-effects model). This is the same as statistical consistency. | - Was the *P*-value for the Q statistic and I^2^ less than a priori defined thresholds (e.g. .10 and 50%, respectively)? - If heterogeneity was detected, was a meta-regression conducted to explain heterogeneity (if sufficient studies)? |
| Robustness (R) | One tests robustness through sensitivity analysis (i.e. removing of one study at a time). The decision on the when to stop should be decided a priori. | - R1: Did confidence intervals of the last three cumulative random-effects meta-analyses remain fully on the same side of zero after (a) removal of the study with the smallest weight, (b) the additional removal of the study with the second smallest weight in the meta-analysis. |
| **Turner-Stokes (2006)**  **Generating the evidence base for the national service framework for long-term conditions: a new research typology** | | |
| ***Domain*** | ***Definition*** | ***Criteria for the domain (converted into signaling questions)*** |
| Design (D) | N/D | - D1: What is the type of the evidence? - Primary research-based (quantitative, qualitative, mixed-methods) - Secondary research-based (meta-analysis; secondary analysis of existing data) - Review-based (systematic review; descriptive or summary reviews of existing research) |
| Study Quality (SQ) | Quality is assessed on the bases of five questions to reach a maximum score of 10. | - SQ1: Are the research question/aims and design clearly stated? - SQ2: Is the research design appropriate for the aims and objectives of the research? - SQ3: Are the methods clearly described? - SQ4: Does the report show a statistically significant and clinically important treatment effect or, for a negative conclusion, have high power? - SQ5: Are the results generalisable? |
| Applicability (A) | Population context of the study. | - A1: What is the population context (people with long-term neurological conditions) of the evidence? |
